# Structural and compositional differences in gallery and spiny forests of Southern Madagascar: Implications for conservation of lemur and tree species

**DOI:** 10.1371/journal.pone.0307907

**Published:** 2024-08-29

**Authors:** Ariadna Mondragón-Botero, Bernard Riera, Hantanirina Rasamimanana, Suzy Razafindrakoto, Eric Warme, Jennifer S. Powers

**Affiliations:** 1 Plant and Microbial Biology, University of Minnesota, Saint Paul, Minnesota, United States of America; 2 Muséum National d´Histoire Naturelle, Paris, France; 3 Ecole Normale Supérieur, University of Antananarivo, Antananarivo, Analamanga, Madagascar; University of Stirling, UNITED KINGDOM OF GREAT BRITAIN AND NORTHERN IRELAND

## Abstract

Madagascar’s unique dry forests, particularly gallery and spiny forests, face severe threats and are significantly understudied, leaving only a fraction of the original extent intact. Thus, there is a critical need for characterizing, conserving, and restoring this diverse forest ecosystem. Conducting extensive floristic surveys and environmental analyses, we investigated structural and compositional differences between the gallery and spiny forests, as well as within distinct gallery forest sites in Berenty Reserve in the south of the island. We also evaluated differences in habitat quality between the spiny and gallery forests for three species of diurnal lemurs in the reserve, and analyzed the current population trend of the tamarind trees, a species of ecological and cultural importance in Madagascar. Our findings revealed that the spiny and gallery forests differed in composition and structure, confirming the unique ecological characteristics of gallery forests and the underexplored richness of spiny forests. Spiny forests exhibited higher species richness despite a comparatively lower sampling effort, emphasizing the need for focused conservation efforts in these overlooked ecosystems. Tamarind populations, vital for lemur nutrition, showed signs of inadequate regeneration suggesting a recruitment bottleneck, possibly due to factors like a lowering water table, brown lemur foraging habits, or shifts in environmental conditions. Urgent interventions, including enrichment plantations, were recommended to ensure the survival of this keystone species. Contrasting botanical and lemur-centric perspectives revealed that while spiny and gallery forests differed botanically, they offered comparable habitat quality for ring-tailed and sifaka lemurs. However, brown lemurs exhibit a preference for the gallery forest, highlighting the intricate relationship between plant composition and lemur habitat choices. Our study underscores the urgency of expanding our knowledge of Madagascar´s dry forests, and Berenty Reserve, as one of the few remaining protected areas with gallery and spiny forests, serves as a reference for future research in Madagascar’s understudied ecosystems.

## 1. Introduction

Madagascar is one of the world´s most important biodiversity hotspots and a critical priority for conservation due to its high species diversity, an exceptional concentration of endemic species, and rapid habitat loss [1, 2]. Most of the biodiversity in Madagascar is concentrated in forests, as 90% of animals on the island are forest dependent. However, Madagascar has lost 44% of its natural forest cover during the past 60 years [3] mostly due to overexploitation, charcoal production, shifting agriculture, and mining [4]. Broadly, the forests in Madagascar can be divided into the moist forests in the east, the mangroves along the west coast, and the dry forests in the west and south of the island. Among Madagascar´s forest ecosystems, the dry forest stands out as the most unique, yet least monitored and protected one [5–7]. Despite a rapid increase in protected areas in Madagascar over the past 20 years [8], the dry forests remain highly underrepresented with less than a third of their total extent under formal protection [9], reflecting global patterns for this ecosystem [10]. Thus, cataloging the diversity that currently exists is a necessary prerequisite for both restoration projects and the conservation of the exceptional biodiversity they comprise.

Dry forests in Madagascar can be further subdivided into dry deciduous forests to the west and spiny forest or spiny thickets to the south [3]. Gallery forests are found within the dry deciduous or spiny forests, growing along river margins, and can differ structurally and compositionally from their surrounding vegetation, adding up to the uniqueness and high species richness of the dry forests. Southern gallery forests in Madagascar are clearly one of the most threatened forest types in the island [11, 12]. Despite the dry forests being almost half of the total forested area in Madagascar [3], most of the research in the dry forest in Madagascar has been focused in the fauna—notably lemurs- and much less research has been centered around the flora or forest ecology [9, 13, 14] (S1 Fig). Therefore, studying the dry forest composition and structure is an important first step towards their conservation and restoration.

**Fig 1:**
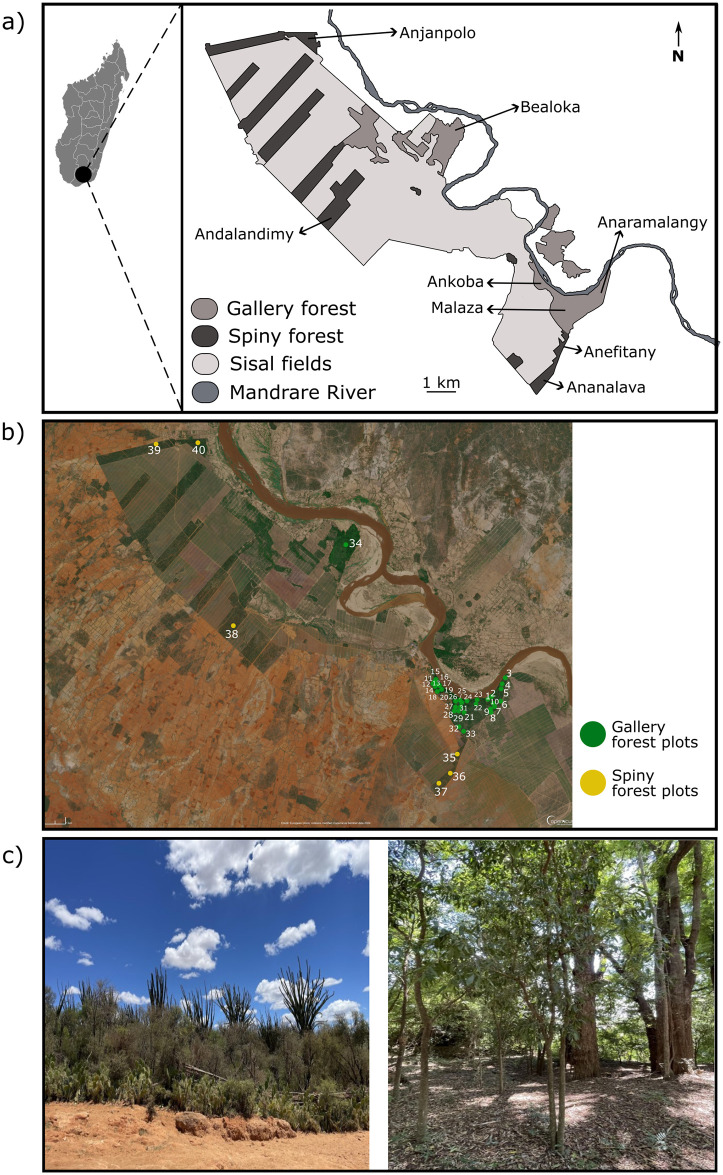
Gallery and spiny forests at Berenty Reserve, Madagascar. **(a)** Forest locations at Berenty reserve (Modified from Rambeloarivony & Jolly 2013b). **(b)** Aerial photograph of Berenty Reserve forests, showing forest plot locations. Spiny forest plots are indicated with yellow circles, and gallery forest plots are indicated with green circles (Image: Modified Copernicus Sentinel data [2024]). **(c)** Photo of the spiny forest, and **(d)** Photo of the gallery forest (Photos: Ariadna Mondragón-Botero).

The Berenty Reserve in southern Madagascar is an excellent place to investigate dry forest characteristics it is one of the few areas that protect the spiny forests in the region, and harbors some of the few gallery forests embedded within the spiny forests, remaining below the headwaters of the Mandrare River (Fig 1). Forest patches in Berenty provide refuge for six species of lemurs, the most threatened mammal group on Earth [15, 16]. Despite its protected status, forests in Berenty are becoming degraded, progressively losing their canopy cover, and are threatened by the invasion of a succulent vine, *Cissus quadrangularis* L. [17, 18]. Alarmingly, over the past 30 years there has been a decrease of the dominant tamarind trees (*Tamarindus indica* L.), which are a key food resource for lemurs [17]. Reduced abundance of this important food resource has already had detrimental consequences for lemur populations [19, 20] and if the trend continues, it will impact the ability of Berenty Reserve to support its dense lemur populations [21]. Thus, forest restoration and conservation of the last remnants of forest in Berenty is crucial for both the tree species diversity and for the lemurs that depend on them [17].

As in other protected areas in Madagascar, the bulk of research at Berenty has been focused on lemurs (S1 Fig). Previous researchers, who most were primatologists, have distinguished three different vegetation zones within the gallery forest in the reserve (Ankoba, Malaza, and Anaramalangy), and have separated the gallery and spiny forest as distinct vegetation types with a sharp ecotone separating both (Fig 1). Various studies have used these distinct vegetation zones to compare lemur populations, and have suggested that lemur population density, troop dynamics, resource quality and availability, feeding behavior, and demography are different among these areas [17, 19, 20, 22–25]. In other words, these areas of the forest differ from a “lemur-centric perspective”. However, if and how these areas of the forest differ botanically has not been tested rigorously.

Therefore, the goals of this study were 1) to determine if and to what extent forest communities differ structurally, or compositionally, between gallery and spiny forest, and within the three gallery forest areas at Berenty 2) to examine whether edaphic factors (soil texture, moisture, and pH) differed consistently among forest types and if those differences helped explain differences among forest areas, 3) to test if the previously designated distinct vegetation zones differ in terms of habitat quality for the three species of diurnal lemurs in Berenty, and 4) to analyze the current tamarind tree population trend in Berenty. Our first hypothesis is that spiny and gallery forests differ in tree species composition and structure. Our second hypothesis is that within the gallery forest, the three forest sites also differ in terms of composition and structure in accordance with previous findings linking lemur biology to the different forest sites. Our third hypothesis is that habitat quality for lemurs differs between the gallery and spiny as well as among the three gallery forest sites. We expect that if floristic and structural differences among the forests in Berenty exist, those differences correspond to different habitat quality for lemurs. Determining if these forest areas comprise distinct plant communities from compositional, structural, or lemur habitat perspectives will inform current and future research on lemurs, and it is crucial to guide and prioritize management and restoration initiatives in the reserve. Lastly, we predicted that tamarind populations show size-class distributions patterns consistent with patterns of decline, wherein larger and presumably older trees are not being replaced by younger trees. Berenty Reserve stands as one of the few areas with spiny and gallery forest fragments in Madagascar, which are rare and threatened throughout the island [5]. Our research in Berenty can serve as a blueprint for similar studies in other regions of Madagascar harboring spiny and gallery forests, providing vital information to advance their conservation and restoration.

## 2. Methods

### 2.1. Research site and history

This study was conducted in the private Berenty Reserve (24°58’60’’S, 46°16’60’’E) in Madagascar (Fig 1). The reserve is located in the Mandrare River Basin, lying on volcanic rocks from the late upper Cretaceous period [26]. The region experiences a dry season from April to September, with nighttime temperatures below 10°C, and a wet season from November to March with midday temperatures sometimes exceeding 40°C [15, 27].

Berenty Reserve was founded in 1936 when the de Heaulme family established sisal (*Agave sisalana* Perrine) plantations along the Mandrare River. Through negotiation with the local Antandroy clans, a total of 1000 ha of forest were preserved, comprising patches of both spiny and gallery forests [28]. The primary gallery forest tract includes three different areas: Ankoba, Malaza, and Anaramalangy (Fig 1). Ankoba, a 40-ha mature second-growth forest was cleared by local Antandroy for farming and agriculture, but since the 1950s it has been allowed to regenerate. Introduced tree species like *Pithecellobium dulce* (Roxb.) Benth., *Cordia caffra* Sond., *or Azadirachta indica* A.Juss. have become an important component of the lemur diet and sustain the highest lemur population density in the reserve. Malaza, a 100-ha gallery forest, has been protected from outside disturbance since 1936, although illegal logging has been reported. Anaramalangy is a 60-ha forest fragment, where no previous plant inventories have been carried out [12, 15, 17]. Bealoka is a120-ha fragment of gallery forest, located to the west of the reserve and was grazed by goats and zebu cattle until 1985 [12]. The spiny forest has a total extent of 1000 ha, divided into eight strips of forest fragments interspersed with sisal plantations. These spiny forest fragments have not been systematically inventoried [25].

### 2.2. Floristic surveys

We established 34 0.1 ha (20 x 50 m) plots in the gallery forest and 6 0.1 ha plots in the spiny forest. Plot locations were stratified by forest site and were positioned to overlap with lemur feeding territories, to ensure our floristic surveys were relevant for habitat quality calculations (Fig 1, S1 Table). Floristic surveys were conducted from June to August 2018 and 2019. In each plot we counted, identified, and measured all the trees that met one of two criteria: a) they had a diameter at breast height (DBH) greater or equal to 10 cm (i.e., 1.3 m from ground level), or b) they had multiple branches below 1.3 m, whose cumulative stem diameters summed more than 10 cm. To compare with recently standardized sampling methods for the dry forest [e.g. 27] we determined the proportion of trees whose combined stem diameters equaled 10 cm. We also assessed how many of these trees met the criterion of having individual stems with a diameter smaller than or equal to 5 cm. In multi-stemmed trees, each stem was measured individually and their basal area was calculated separately and then summed. This ensured that each basal area used in subsequent analyses represented a single tree. We used this method because many species in the spiny forest are short-statured and/or multi-stemmed as adults. Additionally, we established a 1 x 10 m subplot in each plot to identify, count, and measure all individuals with DBH smaller than or equal to10 cm, without a lower DBH limit for inclusion. All individuals were identified to the lowest possible taxonomic level in the field or at the Tsimbazaza Herbarium in Antananarivo (TAN). The accepted botanical names and plant families were adopted from the World Flora Online database [29].

### 2.3. Environmental factors

We measured soil moisture concentration (%), texture, and pH for each plot from ten soil cores (0–30 cm depth). Samples were collected from each corner and six random locations after clearing debris, following at least 6 rain-free days. These measurements were done during the dry season (July–August). Samples were composited by plot, air-dried for 24 hours, and sieved (2 mm). Soil moisture was determined using a soil moisture sensor (SM150T, Delta-T Services) used in the plot, or by the gravimetric method [30]. Fractions of clay, silt and sand in soil were determined using the LaMotte Soil texture test that uses the settling method. Soil pH was measured in distilled water on the air-dried samples using a 1:2.5 soil to solution ratio using a Milwaukee MW102 Portable pH meter with a ±0.02 pH accuracy.

### 2.4. Habitat quality index

We calculated the lemur habitat quality of each site as the sum of the basal area of lemur food tree species. We chose the basal area of lemur food tree species since this metric can give an estimate of food abundance [31]. We obtained information on lemur diets in Berenty Reserve from published articles [22, 32–34]. Two metrics were employed: total basal area per hectare (m^2^ ha^-1^) and relative basal area (%) representing the proportion of basal area occupied by lemur food species relative to the total tree basal area within a plot. Additionally, we calculated primary lemur habitat quality as the sum of the basal area of the primary tree species (i.e., species that constitute > 75% of the lemur diet across different seasons). Both total and relative basal areas were separately used for this calculation This analysis was carried out separately for each of the three diurnal lemur species in Berenty (sifaka: *Propithecus verreauxi*; ring-tailed: *Lemur catta;* brown: *Eulemur fulvus rufus x collaris*) because they have different dietary preferences.

### 2.5. Data analysis

Forest structure metrics encompassing tree density (stems ha^-1^), basal area (m^2^ ha^-1^), and DBH size classes were calculated using data from all trees with a DBH equal to or greater than 10 cm within each 0.1 ha plot. We generated rarefaction and species accumulation curves to compare species richness among samples and evaluate sampling effort [35]. To measure alpha diversity, we calculated Shannon’s diversity index (H’), Simpson’s dominance index (1—D) and Pielou’s index of evenness (J). The Importance Value Index was computed for each forest site at both the species and family level, to compare their relative contribution to forest species composition and assess the differences or similarities between forests. The indexes were calculated as described by Cadotte *et al*. [36]:

$${\text{IVI}}_{\text{f}}\left(\text{family}\right)=\text{relative}\;\text{dominance+relative}\;\text{density+relative}\;\text{diversity}$$

$${\text{IVI}}_{\text{s}}\left(\text{species}\right)=\text{relative}\;\text{dominance+relative}\;\text{density+relative}\;\text{frequency}$$

We compared diversity indices, forest structure metrics, and lemur habitat quality between the spiny forest and the gallery forest, and among the three gallery forest sites. We calculated median values of each of the evaluated parameters for each plot. These median values per plot were then used as individual data points (N = 34 for gallery forest; N = 6 for spiny forest) in our statistical models. Non-parametric Kruskall-Wallis tests were used for testing differences followed by a Dunn´s test if significant differences were found. We created a Venn diagram to depict species number and overlap between the two forest types or across the gallery forest sites. We used a non-metric multidimensional scaling (NMS) ordination based on Bray-Curtis dissimilarity to visualize the differences between the three forest types or sites in terms of species composition. To test the relationship between species composition and environmental variables, a distance-based redundancy analysis (db-RDA) with Bray-Curtis distances was conducted [37]. Prior to the analysis, Pearson correlation tests were performed to ensure the independence of the environmental variables. Only variables that differed statistically (p <0.05) between either forest types or forest sites were used as constraining factors. Variables were transformed if needed by taking their square root to avoid negative eigenvalues. A permutation test was performed to test the significance of the analysis and of each variable. Diversity analyses, constrained ordination analysis and species accumulation curves were performed using the Biodiversity R [38] and vegan packages [39]. We performed an individual-based rarefaction and extrapolation to compare species richness between forest sites while accounting for differences in the number of individuals sampled [40]. Additionally, we were particularly interested in evaluating tamarind regeneration. Our study prioritized tamarind trees based on their ecological importance as a major food source for lemurs and their dominance within the gallery forest ecosystem [11]. This emphasis is further supported by the lack of comparable data on regeneration challenges for other potential keystone species in the Berenty Reserve. Therefore, we analyzed tamarind tree densities and frequency distribution across all plots and subplots, and compared visually the number of seedlings versus adult trees to identify any potential trends in tamarind populations. All statistical analyses were conducted in R [41].

### 2.6. Ethics statement

This research did not involve direct data collection from human subjects or live animals. The analysis presented is based entirely on previously published data and did not require the direct involvement of animal or human participants. Therefore, Institutional Review Board (IRB) approval or ethical review was not applicable for this study. All data sources are appropriately cited within the manuscript, adhering to the ethical standards for secondary data analysis.

## 3. Results

### 3.1 Floristic composition and diversity

We identified a total of 2975 tree individuals, belonging to 60 genera and 31 families. Of these, 75 taxa were identified to species level while 8 were identified to the genus level (S2 Table). Species richness (calculated as the number of species in each forest type) differed significantly between the gallery and spiny forests (X^2^ = 38.89, p<0.05). The spiny forest had a higher species richness despite our comparatively lower sampling effort. Species richness, as assessed by rarefaction and extrapolation curves, was higher in the spiny forest than in the gallery forest at equivalent numbers of individuals (S2 Fig). Species richness was also different among the three distinct gallery forest sites (X^2^ = 6.17, p<0.05). Moreover, species composition significantly differed between the spiny and gallery forest, with only 10 species common to both forest types, separating these two into two distinct forest types (S2 Table). The non-metric multidimensional scaling followed by a PERMANOVA also showed that spiny forest plots formed a separate cluster from the gallery forest plots, indicating that gallery and spiny forests were differentiated based on their species composition (S3 Fig).

**Fig 2:**
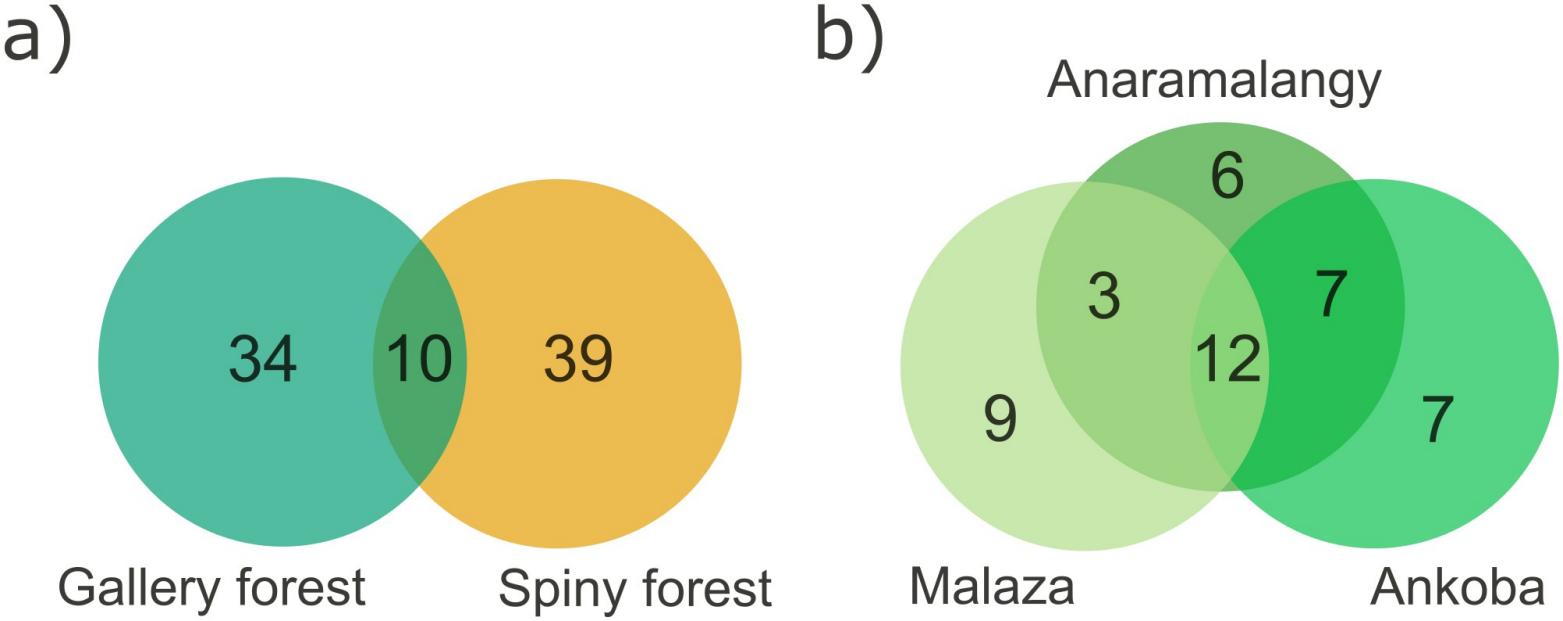
Number and overlap in species in the different forest areas in Berenty Reserve. The Venn diagrams show the overlap between **a)** the gallery and the spiny forest, and **b)** among the three sites of the gallery forest. Only 10 species were shared between the spiny and gallery forests, and 12 were common to the three gallery forest sites.

**Fig 3:**
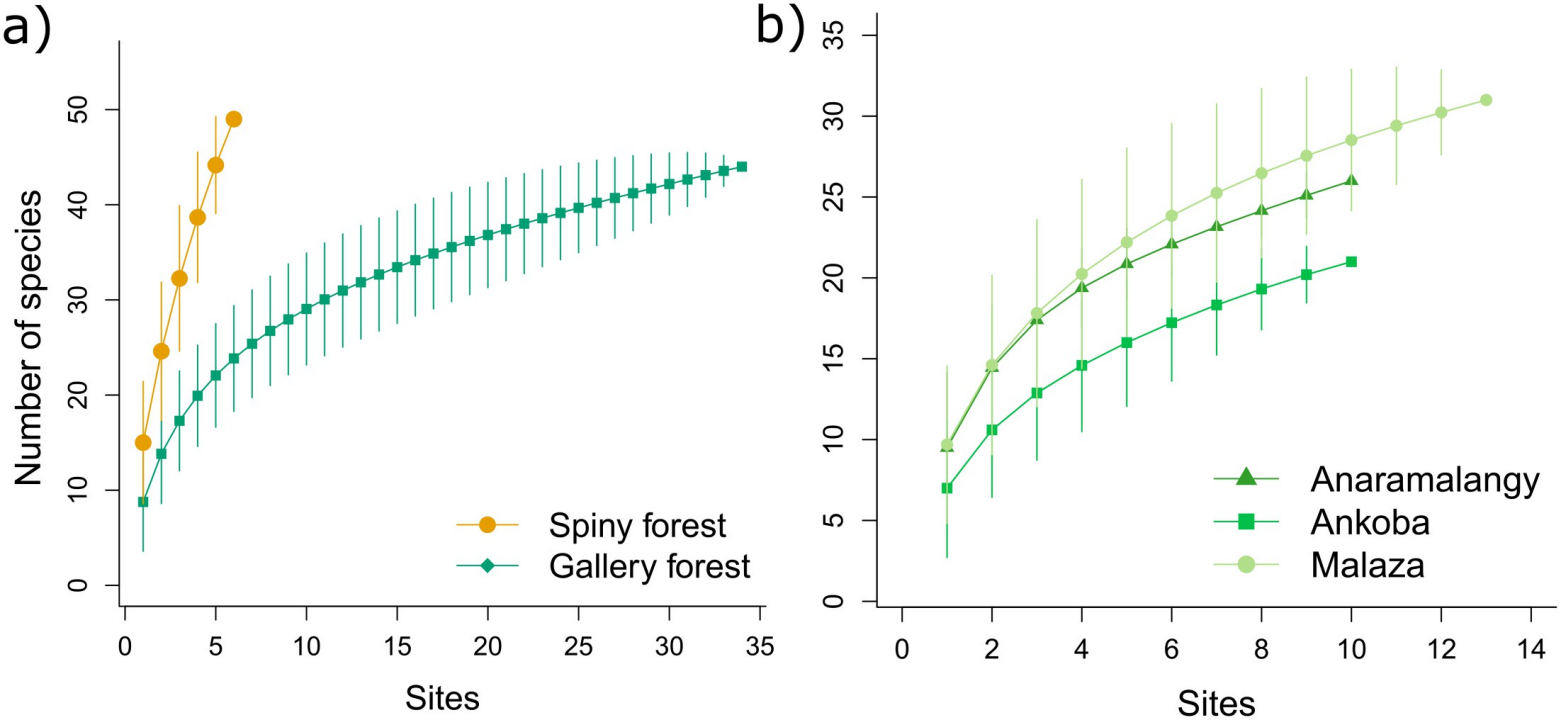
Species accumulation curves for the spiny and gallery forests in Berenty Reserve. **a)** gallery and spiny forests where the yellow line (dots) corresponds to the spiny forest and the green line (squares) corresponds to the gallery forest; **(b)** Species accumulation curves for the three different within the gallery forest areas: Anaramalangy (triangles), Ankoba (squares), and Malaza (circles). The x-axis represents cumulative sampling effort, with each point corresponding to an additional sampling site (or plot) surveyed. Vertical lines on each curve show the 95% confidence interval.

Species richness and composition were also significantly different among the three gallery forest sites with only 12 species shared across them (Fig 2; S2 Table). The NMDS analysis showed that this variation stemmed from the distinct separation of six plots in Ankoba, while Anaramalangy and Ankoba plots clustered together (S3 Fig). Despite harboring different species, none of the calculated diversity indices differed significantly between spiny and gallery forests (Table 1) or among the three gallery forest sites (Table 2). Additionally, none of the species accumulation curves reached an asymptote (Fig 3).

**Table 1:** Comparisons of diversity, structure, and environmental variables between the gallery and spiny forests. Asterisks denote significant differences between forest types assessed with a Kruskal-Wallis test. Different letters (a, b) indicate significant differences between the two forest types assessed via a Dunn’s test.

Variable	Gallery Forest	Spiny Forest	X^2^	P-value
Richness	44 ^a^	49 ^b^	38.89	<0.05
Shannon Diversity Index	1.57 (0.06) ^a^	1.69 (0.23) ^a^	0.69	0.41
Simpson Dominance Index	0.69 (0.02) ^a^	0.67 (0.08) ^a^	0.28	0.59
Pielou’s Evenness Index	0.74 (0.02) ^a^	0.63 (0.07) ^a^	1.76	0.18
Density (trees/ha)	580 (42.22) ^a^	1600 (288.54) ^b^	13.79	<0.05
Basal area (m^2^/ha)	25.5 (1.80) ^a^	40.6 (6.52) ^b^	3.58	<0.05
pH	7.94 (0.09) ^a^	7.08 (0.14) ^a^	8.74	<0.05
Moisture	9.77 (0.60) ^a^	3.46 (1.17) ^b^	10.61	<0.05
Sand (%)	49.85 (2.11) ^a^	61.11 (2.42) ^b^	4.14	<0.05
Silt (%)	38.23 (2.22) ^a^	33.05 (1.90) ^a^	1.66	0.20
Clay (%)	12.10 (1.02) ^a^	7.51 (1.59) ^a^	2.31	0.13

**Table 2:** Comparisons of diversity, structure, and environmental variables among the three gallery forest areas. In a row, asterisks and different letters indicate significant differences between forest types assessed with a Kruskal-Wallis test. Different letters (a, b, c) indicate distinct groups identified by a Dunn’s test.

Variable	Anaramalangy	Ankoba	Malaza	X^2^	P-value
Richness	26 ^a^	21 ^b^	31 ^c^	6.17	< 0.05
Shannon Diversity Index	1.66 (0.11) ^a^	1.42 (0.13) ^a^	1.67 (0.09) ^a^	2.61	0.27
Simpson Diversity Index	0.73 (0.03) ^a^	0.67 (0.05) ^a^	0.72 (0.03) ^a^	1.04	0.59
Pielou’s Evenness Index	0.75 (0.03) ^a^	0.75 (0.75) ^a^	0.74 (0.03) ^a^	0.3	0.86
Density (trees/ha)	601 (41.83) ^a^	511 (54.31) ^a^	537 (97.97) ^a^	2.26	0.32
Basal area (m^2^/ha)	23.62 (2.73) ^a^	32.92 (2.42) ^b^	20.75 (3.39) ^ac^	8.8	< 0.05
pH	8.18 (0.08) ^a^	7.62 (0.22) ^a^	8.07 (0.13) ^a^	5.36	0.07
Moisture	7.53 (0.59) ^a^	12.99 (0.65) ^b^	9.43 (0.98) ^ac^	12.8	< 0.05
Sand (%)	49.73 (4.75) ^a^	43.99 (2.51) ^a^	56.46 (3.24) ^a^	5.63	0.06
Silt (%)	37.33 (4.25) ^a^	43.83 (2.56) ^a^	32.82 (3.65) ^a^	3.5	0.17
Clay (%)	12.93 (2.30) ^a^	13.17 (2.01) ^a^	11.23 (1.26) ^a^	0.39	0.82

**Fig 4:**
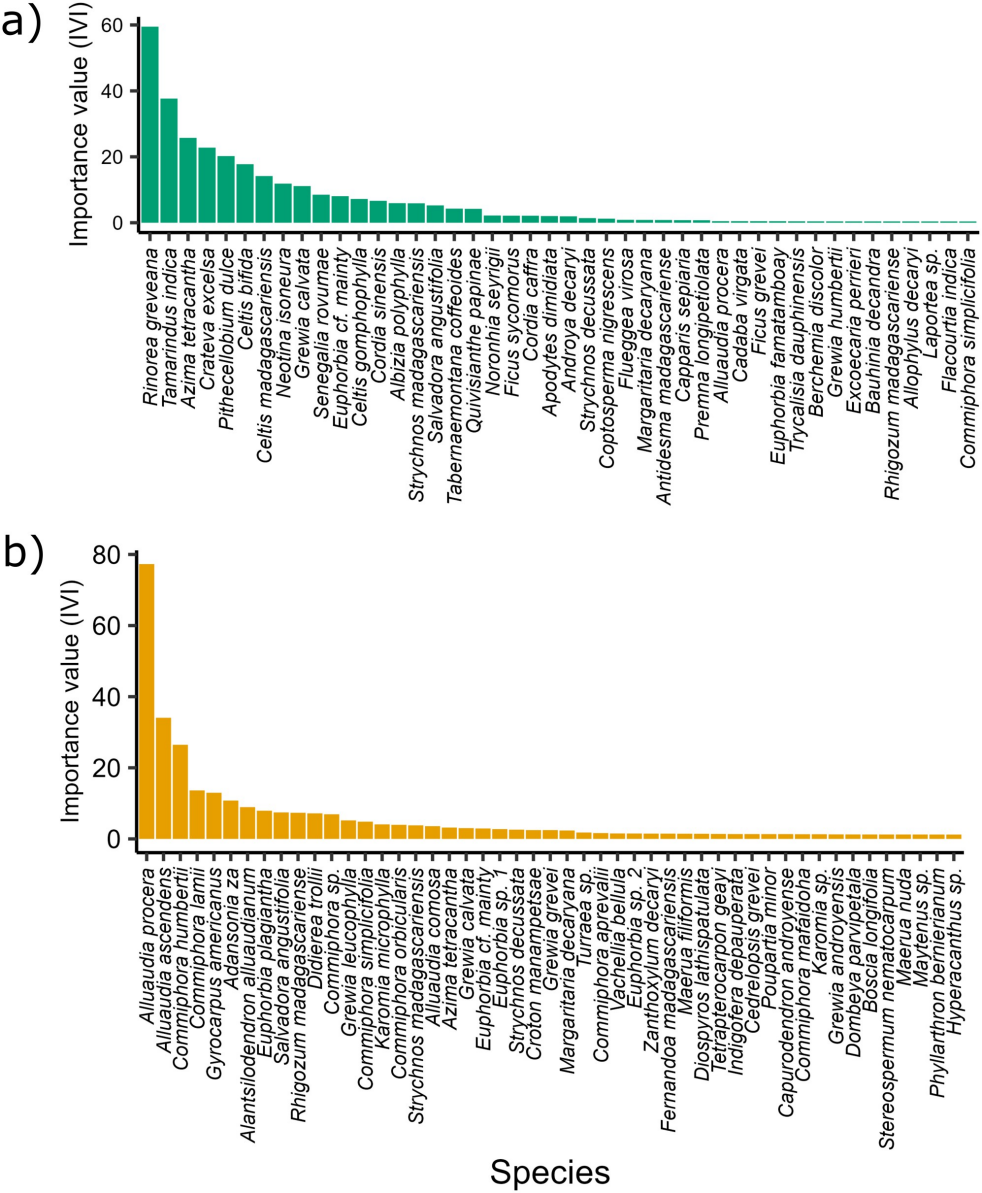
Species Importance Value Index (IVI_s_) for the gallery and spiny forests. The IVI_s_ is shown in **(a)** for the gallery forest and in **(b)** for the spiny forest.

**Fig 5:**
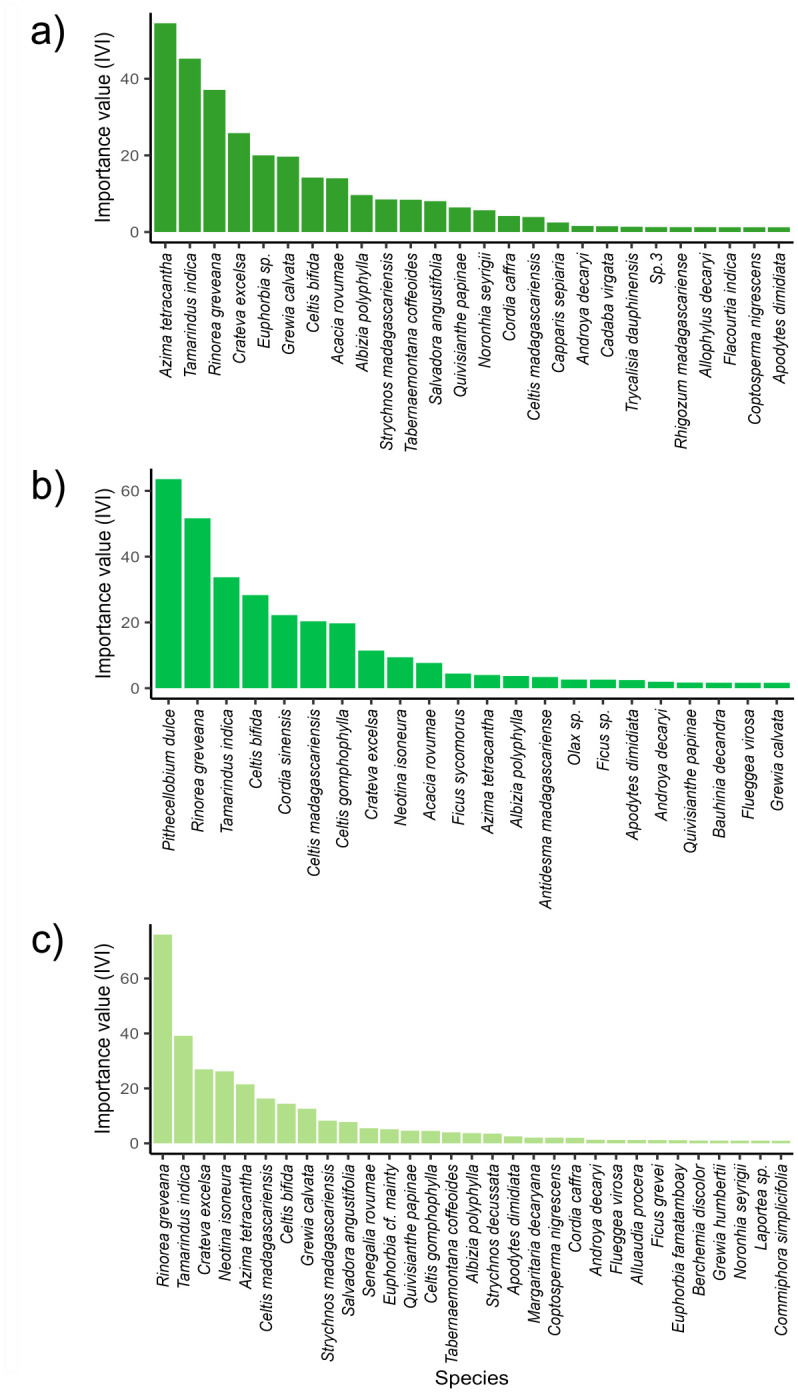
Species Importance Value Index (IVI_s_) the three forest sites within the gallery forest. **(a)** Anaramalangy, **(b)** Ankoba, and **(c)** Malaza.

The dominance of species differed between the spiny and gallery forest. In the gallery forest, *Rinorea greveana* Baill. Was the species with the highest importance value index (IVI_s_) (59.4), followed by *T*. *indica* (37.6), and *Azima tetracantha* Lam. (25.7). In the spiny forest, the three species with the highest IVI_s_ value were *Alluaudia procera* Drake (77.3), *Alluaudia ascendens* Drake (34.1), and *Commiphora humberti* H. Perrier (26.4) (Fig 4). Within the gallery forest, the dominance of species differed among sites. In Ankoba, *P*. *dulce* was the species with the highest IVI_s_ value (63.5), followed by *R*. *greveana* (51.6), and *T*. *indica* (33.7). In Malaza, the three species with the highest IVI_s_ value were *R*. *greveana* (75.9), *T*. *indica* (39.1), and *Crateva Excelsa* Bojer (26.9).. Finally, in Anaramalangy these were *A*. *tetracantha* (54.4), *T*. *indica* (45.2), and *R*. *greveana* (37.1) (Fig 5). A detailed description of the IVI for families (IVI_f_) is provided in S4 Fig.

### 3.2. Forest structure

Diverging significantly from the gallery forest, the spiny forest had a 2.7-fold greater tree density and a 1.5-fold larger basal area (Table 1). The frequency distribution of diameter size classes showed that for both forests, most trees had a DBH between 10 and 70 cm. However, larger trees (with a DBH ≥ 100 cm) were relatively more frequent in the gallery forest compared to the spiny forest (S5 Fig). Plots within the gallery forest had significantly different basal area values, but they did not differ in stem density (Table 1). In total, we measured 1039 individuals in the spiny and 1936 individuals in the gallery forests. In the spiny forest, 12.5% (n = 130) were multi-stemmed trees whose total summed diameters was ≥ 10 cm, and 2.3% (n = 24) were multi-stemmed trees with at least one stem that was ≥ 5 cm DBH. In the gallery forest 24% (n = 471) were multi-stemmed trees whose total summed diameters were ≥ 10 cm, and 2.1% (n = 42) were multi-stemmed trees with at least one stem that was ≥ 5 cm DBH.

### 3.3. Edaphic factors as drivers of forest differences

Gallery forest plots had a significantly higher soil pH and moisture than spiny forest plots (Table 1). Soil moisture was 2.8 times higher in the gallery forest. Soils in both forest types had high percentages of sand and silt, with a low clay content. However, only sand significantly differed between these forest types, being 1.2 times higher in the spiny forest. Soil pH and texture did not significantly differ among the three gallery forest sites, but moisture content was significantly higher in Ankoba compared to Malaza and Anaramalangy. The db-RDA analysis showed that moisture and pH were two factors associated with the differentiation of the gallery and spiny forest plots into two separate clusters. Both factors however only explained 18% (R^2^ adjusted) of the observed variation (F = 5.21, P = 0.001, 999 permutations). Within the gallery forest, the db-RDA analysis showed that soil pH, moisture, and sand percentage explained 20% (R^2^ adjusted) of the variation, separating Ankoba plots into a distinct cluster (F = 2.33, P = 0.001, 999 permutations) (S7 Fig).

### 3.4. Tamarind population

**Fig 6:**
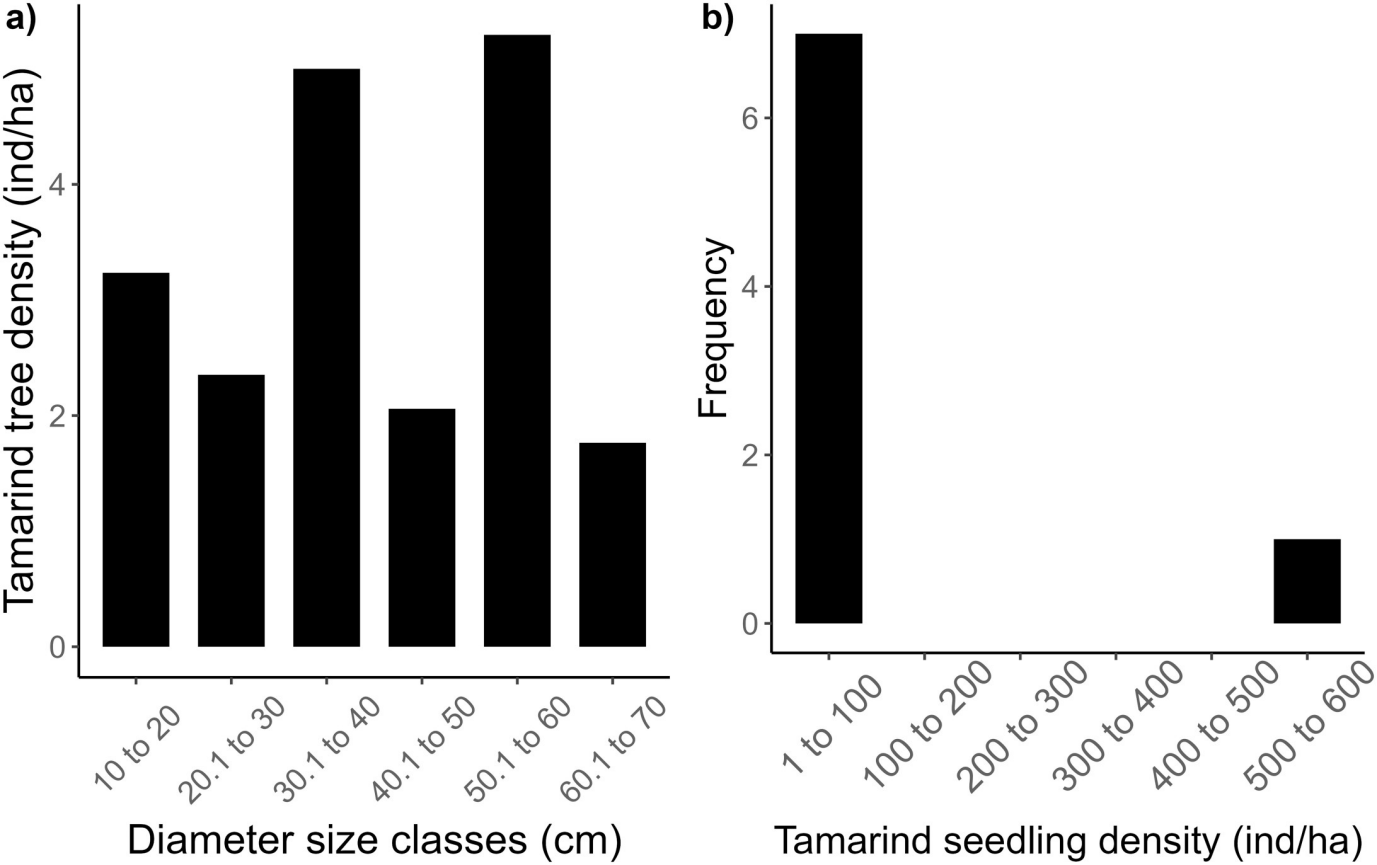
Distribution of adult and seedling tamarind densities in the gallery forest at Berenty Reserve. **(a)** Density of adult tamarind trees in each diameter size class across all the gallery forest plots **(b)** Number of subplots (frequency) per seedling density category. Each category represents the number of tamarind seedlings per hectare within that range (e.g., ‘1 to 100’ indicates subplots where the seedling density was between 1 and 100 individuals per hectare). In this study, we defined adult trees as tamarinds with a DBH ≥ 10 cm and seedlings as plants with diameter at root collar ≤ 1 cm.

We sampled 117 individual tamarinds within the gallery forest plots, including 28 seedlings that were sampled in the subplots (seedlings were defined as plants with diameters < 1 cm). Interestingly, we did not find any individuals within the DBH range of 2 to 10 cm in any of the subplots. Tamarind seedlings were only found in 8 out of the 41 subplots, showing a clumped or patchy distribution. In 7 of these subplots the seedlings had a density of up to 100 individuals per ha and only one plot had a tamarind seedling density of > 500 individuals per ha (Fig 6a and S8 Fig). We did not find contrasting differences in soil pH or moisture content between the plots with tamarind seedlings (mean pH 8.01, moisture content 10.68%) and those without (mean soil pH 7.91 and moisture content 9.48%). For adult tamarind trees, the majority of individuals in our sample had diameters at breast height ranging from 30 to 60 cm, and we saw no evidence for the “reverse J” pattern of densities against size class where smaller sized individuals are more abundant than larger diameter individuals (Fig 6a). Of the 34 sampled subplots in the gallery forest, most (n = 28) contained no tamarind seedlings, and only one subplot fell into the “500 to 600” density category, indicating a much rarer occurrence of high seedling densities (Fig 6b). Plotting of the distribution of tamarind seedling density against adult tamarind density in the gallery forest plots showed that many plots did not have any seedlings in the understory despite having adult tamarind trees. Even in plots with the highest adult densities, zero seedlings were observed (S8 and S9 Figs).

### 3.5. Habitat quality index

**Fig 7:**
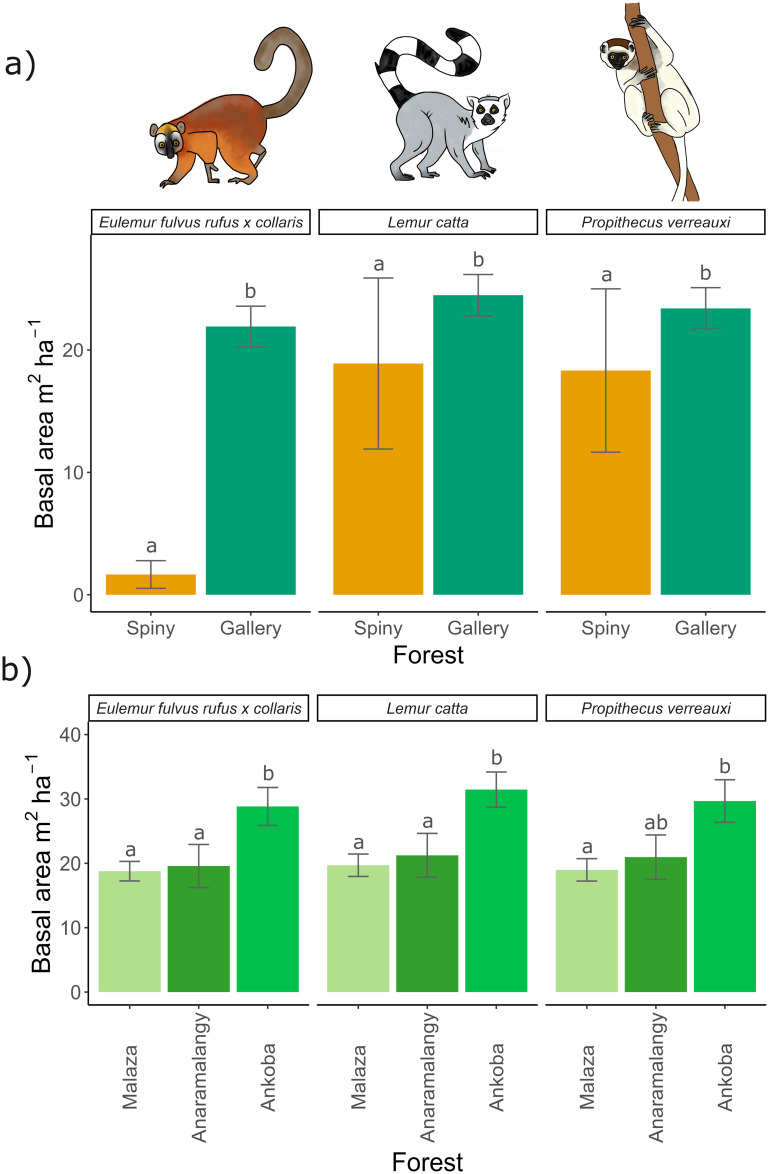
Lemur habitat quality in Berenty Reserve. The habitat quality is represented as the total basal area of all food tree species consumed by the three species of diurnal lemurs in **(a)** the gallery and spiny forests, and **(b)** the three different gallery forest sites. Comparisons are done within lemur species. Vertical bars represent standard errors and different letters indicate significant differences between forests.

There were no significant differences in basal area of food trees for lemurs between the spiny and gallery forests, except for brown lemurs. The basal area of food trees was 6 times higher in the gallery forest (22.4 m^2^ha^-1^) than the spiny forest (2.1m^2^ha^-1^) for this species (Fig 7 and S10 Fig). When considering primary food trees for lemurs, we found a substantial difference between the gallery and spiny forest plots, notably for sifaka and brown lemurs (S10 Fig). Gallery forest plots had a higher habitat quality index for these two species. For ring-tailed lemurs, although not statistically significant, the total basal area of primary food trees was 68% higher in the spiny (17.8 m^2^ha^-1^) than in the gallery forest (10.6 m^2^ha^-1^). Within the gallery forest, Ankoba notably exhibited a significantly higher basal area of food tree species than Malaza, a pattern consistent across all three lemur species (Figs 7 and S10). Similarly, Ankoba had a significantly higher basal area than Anaramalangy, except for the sifaka lemur. Concerning primary food tree species, Ankoba had a significantly higher basal area compared to Malaza and Anaramalangy for sifaka lemurs. However, for the brown and ring-tailed lemurs, the basal area of the primary food tree species was not significantly different among the three gallery forest sites (S11 Fig).

## 4. Discussion

Our study represents the largest forest inventory in Berenty Reserve to date, and the first comprehensive assessment of the plant composition and diversity of both the spiny and gallery forests. Overall, our results highlight the marked differences between these two forest types, both from a botanical and a lemur-centric perspective. Importantly, spiny forests have a larger species richness and a unique, yet underexplored flora. These findings underscore the need for tailored conservation and restoration efforts in both forest types with an increased attention to the spiny forest. The three gallery forest sites, while showing some variation, did not differ significantly in terms of diversity or structure, and only a few forest plots in Ankoba differed in terms of composition. Likewise, from a lemur-centric-perspective only Ankoba differed in terms of habitat quality. In light of these findings, we suggest that the three forest sites may be treated as a gallery forest continuum along the Mandrare River rather than distinct forest types. Furthermore, our study stresses the urgency of restoration interventions for the tamarind tree, a keystone species supporting lemur populations in Berenty. Collectively, our research not only deepens our understanding of forest fragments in Berenty but also provides valuable insights for conservation and restoration efforts in similar habitats across Madagascar. Below we discuss our findings in light of both tree and lemur conservation at Berenty.

### 4.1. Spiny and gallery forests are distinct

Confirming our hypothesis, the gallery and spiny forests were different in terms of floristic composition and structure. The presence of species unique to either the spiny or gallery forests underscores their conservation importance and emphasizes the need for tailored conservation and management strategies. For example, for restoration efforts, identifying these distinct species in each forest type can guide the selection of species that are best suited for particular environmental conditions, thereby increasing the chances of restoration success. Gallery forests, known for their ecological uniqueness, often exhibit marked differentiation from their surrounding landscapes, frequently characterized by sharp ecotones [5, 42]. This pattern is evident in Berenty, where the gallery forest vegetation is maintained by a higher water table that drastically decreases with increasing distance and elevation from the river [18]. Soil characteristics have been widely identified as a major driver of plant species assemblages [43]. In our study, edaphic factors such as pH and sand concentrations stood out as key factors delineating the plant communities in spiny and gallery forest, although the variance explained by these factors was very modest.

Notably, the spiny forest exhibited higher species richness than the gallery forest, despite our sampling effort being comparatively lower in the former. Moreover, our species accumulation curves demonstrated that neither forest type had reached an asymptote, indicating that further botanical exploration may unveil even more plant species within these ecosystems. It is worth noting that our criteria for sampling trees slightly differed from other methods. However, if our sampling had exclusively targeted individuals with at least one stem equal to or greater than10 cm DBH, we would have inadvertently omitted 20% of the recorded individuals within our inventory. Given that nearly all of these individuals possessed at least one stem with a diameter of 5 cm or more at breast height, we recommend that future sampling efforts should consider an expanded focus on trees with stems measuring at least more than 5 cm DBH to comprehensively capture the full spectrum of forest diversity, and to mitigate the risk of underestimating spiny forest cover [7, 44]. Lastly, the lower basal area and tree density in the gallery forest compared to the spiny forest may be attributed to the prevalence of multi-stemmed trees in the spiny vegetation, and to the densely occurring individuals of the Alluaudia and Didierea genera. These differences highlight the importance of our sampling approach for accurately capturing forest diversity.

While the gallery forest has historically received most of the attention in Berenty largely due to accessibility and the imminent risk of its disappearance, our study underscores the need for increased attention to the spiny forest. This forest type harbors a rich but threatened biodiversity. Indeed, two of the most important species in the spiny forest were classified as Vulnerable (*Alluaudia ascendens*) or Near Threatened (*Commiphora lamii* H. perrier). Both species are characteristic of and endemic to the spiny thickets. *A*. *ascendens* is an important food resource for lemurs and bats [45]. The loss of such species would mean the irreversible loss of unique genetic information and of the ecosystem services they provide. Accordingly, we recommend focused research in several key areas such as the study of regeneration of at-risk species, or ecophysiological traits that confer resilience to environmental stressors, to support restoration efforts. Research on the anthropogenic impacts on the spiny forest is also necessary to understand how human activities influence its ecological integrity [6]. Given the extensive fragmentation of the spiny forest in Berenty, as is the case for other spiny forests in Madagascar [46], conservation efforts should prioritize fragment connectivity, especially for endemic and vulnerable species with a limited distribution.

Given the differences found between the gallery and spiny forest, our results highlight the need for specific conservation efforts recognizing the unique ecological roles of both forest types in preserving the region’s rich biodiversity. Likewise, our study demonstrates the importance of conducting fine scale studies of the gallery and spiny forests that can be replicated in other dry and gallery forests in the rest of the island, where baseline data on forest composition, regeneration, and succession processes on these ecosystems are urgently needed [5].

### 4.2. A continuous gallery forest

We initially hypothesized that there would be substantial variation in plant composition among the three forest sites within the gallery forest. Contrary to our expectations, our analysis revealed that most of the plots shared a high degree of similarity. Consequently, from a forest management and restoration perspective, these forests should be regarded as a continuous and interconnected gallery forest patch. However, there were some species that were found exclusively in only one of the forest sites. This exclusivity may stem from either the species’ rarity or a sampling artifact where individuals were not sufficiently large to meet the inclusion threshold in our inventory. For example, *Excoecaria perrieri* Leandri, was a rare species found only at Anaramalangy. Such rare species deserve great attention and should be taken into account in restoration planning of the gallery forest. On the other hand, species like *Cadaba virgata* Bojer or *Tricalysia dauphinensis* Ranariv. & De Block were recorded only in Anaramalangy because they met the inclusion criteria, although we have visual records of them in Malaza and Ankoba. These observations reinforce the need for future studies that include individuals with a DBH smaller than 10 cm to provide a more comprehensive understanding of forest diversity.

Within the gallery forest, a distinct group was formed by six out of ten vegetation plots from Ankoba. Some particular non-native species were restricted to Ankoba in these plots (i.e. *Pithecellobium Dulce* and *Cordia Sinensis*). We suggest that the few statistically significant differences found between Ankoba and both Malaza and Anaramalangy may be driven by the few plots where these non-native species were dominant. Regardless, our findings collectively indicate that the three forest sites are not sharply differentiated in terms of species composition, diversity or structure.

### 4.3. Tamarind decline

Tamarinds are one of the most important and dominant species in Madagascar´s dry and gallery forests [11]. They hold significant ecological, cultural, and economic value in southern Madagascar. Besides being a key food resource for lemurs, tamarinds have positive effects on soil organic carbon, nitrogen, and microbial activity [47]. Additionally, they promote the Oxalate-Carbonate Pathway indicating a high potential to enhance carbon sequestration in the soils [48]. Although it is a pantropically cultivated species, tamarinds are facing a worrying decline in their native and wild range, including in Berenty and other African forests [17, 21, 49–52]. Our results amplify this concern, by showing that tamarind seedlings and saplings are not abundant in Berenty´s forests, and that populations are not regenerating. The low number of adult individuals in the smaller size classes suggest that tamarind populations might not be self-sustaining in Berenty. Additionally, even in plots with the highest adult densities, we did not observe any seedlings or saplings. Most of the tamarinds we found were newly germinated seedlings with typically low survival rates in Berenty´s gallery forests [17, 21]. The absence of individuals within the diameter range of 2 to 10 cm suggests a recruitment bottleneck during a specific period or potential challenges faced by tamarind seedlings and saplings within this size range. This observation aligns with previous findings in Berenty and with the broader context of forest dynamics, where the establishment phase for seedlings is a survival bottleneck [17, 18, 53].

The reasons for tamarind decline in Berenty have been speculated for a long time without reaching a consensus. One significant factor could be a lowering water table, leading to drier soils in the gallery forest [17]. The Mandrare Riverbed, along which the gallery forest grows, is dynamic which may have caused significant shifts in the water table. Additionally, the recently introduced brown lemurs consume tamarind fruits while still green, impacting tamarind seedling recruitment [54]. Shifts in environmental conditions in Berenty, potentially driven by climate change, are also a plausible explanation for the observed tamarind decline. Although rainfall patterns over the past seven decades appear somewhat stable, there are no records before 1948 [18]. Possibly the adult trees in our inventories could have established under environmental conditions different from the present that were more suitable for their establishment. Therefore, it is critical to understand the ideal conditions for tamarind establishment, to elucidate the reasons for tamarind decline across its native range.

Part of the challenge in understanding tamarind decline is a fundamental lack of knowledge concerning its life history strategy. Previous studies suggest that tamarinds are light demanding, characteristic of pioneer species, and were found to be primarily concentrated along open forest paths [17]. However, tamarinds are long-lived trees with live spans of up to 400 years [18], and have slow growth rates in their sapling stage [17, 55], which complicates their classification into a particular ecological role. Interestingly, we recorded fewer tamarind individuals compared to earlier studies, despite our study being the most extensive forest inventory to date. This could be an effect of our sampling design which purposely avoided forest paths and roads, but it could also reflect the diminishing tamarind population in Berenty Reserve. Consistent with previous studies, we found that both adult and seedling tamarinds have patchy distributions and are restricted to certain areas in the gallery forest, which point towards very specific environmental requirements during their seedling and sapling stages [17]. We recommend that future studies focus on elucidating the barriers to tamarind establishment, and to specifically test the resource requirements (i.e. light, water, nutrients) to develop appropriate conservation and restoration strategies for this species in Berenty and in other areas in Madagascar. If tamarind regeneration does not occur at the same pace as the death of older individuals, the populations of this tree and the lemurs and other species that depend on it might be imperiled. Furthermore, given that tamarind trees are one of the dominant species in the gallery forest, their decline could have potential impacts on soil health, carbon sequestration, and overall forest structure. Our results emphasize the urgency of interventions through enrichment plantations of this species in the gallery forest, to ensure that enough young trees will replace the older ones.

### 4.4. Lemur-Centric versus botanical perspectives on forests

While botanical analyses showed a substantial difference in plant composition and diversity between the spiny and the gallery forest, our study revealed a different narrative from a lemur food-centric perspective. The spiny and gallery forests are not different in terms of habitat quality for the ring-tailed or the sifaka lemurs, both offering equal foraging and feeding resources for both species. However, an exception emerged when considering the brown lemur whose habitat quality index was higher in the gallery forest. Although there are territorial disputes over feeding territories, the occupation of different areas could facilitate the long-term coexistence of Berenty’s three diurnal lemur species [54]. Given that food availability is undoubtedly one of the most important factors that determine the distribution, abundance, and behavior of lemurs as well as of other primates [31, 56], our findings might explain why the brown lemurs are currently found exclusively in Berenty’s gallery forest [54]. Furthermore, our findings underscore the intricate relationships between plant composition and lemur habitat utilization.

In terms of primary food tree species, we observed a difference in habitat quality for the sifakas and ring-tailed lemurs between the gallery and spiny forest. The gallery forest offers a higher habitat quality for both species. In Berenty there is a five-fold difference in ring-tailed lemur density ranging from about 500 individuals/km^2^ in Ankoba to about 100 individuals/km^2^ in the spiny forest [12, 57] (S3 Table). Additionally, ring-tailed body biomass is greater in the riverfront forest than in other parts of the reserve [20]. Similarly, there is a nine-fold difference in sifaka lemur density from about 956 individuals/km^2^ in Ankoba to about 140 individuals /km^2^ in the spiny forest (S3 Table). These differences in habitat quality, lemur density, and body composition between the spiny and gallery forest might in part be attributed to the presence of tamarind trees, which provide a rich protein source for lemurs. Similar results regarding the importance of tamarind were found in Bezà Mahafaly Reserve when contrasting lemur densities and availability of food species between the spiny and gallery forest [58]. Similarly, *Rinorea greveana*, the species with the highest IVI in the gallery forest, is absent from the spiny forest. This species is among the most frequently consumed tree species by lemurs, and thus likely contributes to the higher habitat quality of the gallery forest for lemurs compared to the spiny forest.

Ankoba, within the gallery forest, consistently exhibited higher basal areas of food tree species for all three lemur species. This distinction aligns with both botanical and lemur-centric perspectives, as Ankoba also differs significantly in its plant composition compared to the other forest sites. Ankoba exhibits the highest sifaka (956 individuals/km^2^) and ring-tailed 427 individuals/km^2^ lemur densities compared to Malaza (sifaka: 199 individuals/km^2^; ring-tailed 171 individuals/km^2^) and Anaramalangy (sifaka: 167 individuals/km^2^; ring-tailed: 60 individuals/km^2^) [57]. Ankoba’s distinction from both a botanical and a lemur perspective might be attributed to the presence of certain non-native species, particularly *P*. *dulce* and *C*. *sinensis*, known for their high protein content [33]. It is important to acknowledge that while non-native plants can be an important food source for lemurs, they can also lead to health issues such as alopecia seen in lemurs that consumed *Leucaena leucocephala*, which has now been removed from the reserve [59]. Our findings not only stress the importance of fine-scale habitat assessments, but it also suggest that patch quality plays a pivotal role in maintaining primate populations in fragmented environments, often more so than patch size alone [60]. Additionally, it is it is important to recognize that other factors such as forest structure including tree height, DBH, or density can influence lemur distributions in Berenty’s forests [61, 62]. Future research should further explore these and other relevant aspects to have a more comprehensive understanding of lemur habitat quality and lemur distributions.

### 4.5 Placing Berenty Reserve in the regional context

Diversity and forest structure metrics from Berenty’s forests lie within the values found for other dry forests in Madagascar. Tree densities in other studies have been reported to range from 775±223 stems/ha in Kirindy Mitea National Park [63] to 3000 stems/ha in Beza-Mahafaly [11]. Basal area values found in the gallery forest in our study site are similar to those reported for the gallery forests in Andohahela (25,1 ± 13,8 m^2^/ha) [62]. However, basal area values found our study for the spiny forest are lower compared to those in the spiny forests in Andohahela (96,4 ± 34,1 m^2^/ha) which may be attributed to their inclusion of trees with a lower DBH limit of 2.5 cm [62]. In Berenty, a few species largely dominate the forest plots, both in the gallery and spiny forests. This pattern is consistent with many tropical forests [64] that show a general pattern of oligarchic dominance, with a very few common species and a large number of rare taxa [65, 66].

The families with the highest IVI values in our study area are also among the most important ones in other tropical dry forests in Madagascar, and the Neotropics [67]. Notably, Fabaceae was the most important family in the gallery forest, similar to findings in several studies conducted in the Neotropics [68, 69]. On the other hand, we found that the iconic Didieraceae family was the most important one in the spiny forest. All the species in this family belong to the subfamily Didiereoideae which is endemic to the southwest of Madagascar, and is characteristic of the spiny thickets.

Berenty´s forests are also comparable in species richness to those of Beza-Mahafaly, with 42 reported species in the gallery and 46 reported species in the spiny forest [11]. However, this richness is lower than that of Tsimanampetsotsa National Park, where 63 species have been reported in the spiny forest [70]. Some of the most common species like *Tamarindus indica*, *Gyrocarpus americanus* Jacq., *Acacia rovumae* (Oliv.) or *Azima tetracantha* are common to both Beza-Mahafaly and Berenty, and several genera are common as well. Nevertheless, distinctions emerge at the species level, emphasizing the marked spatial differentiation and species specificity inherent to Madagascar’s dry forests. This has significant implications for their restoration and conservation. Precisely identifying the specific species within a region is crucial for establishing reference ecosystems essential for restoration efforts, and at the same time highlights the importance of detailed studies in other spiny and gallery forests across Madagascar [5].

Berenty exhibited remarkably high lemur densities compared to other reserve areas in Southern Madagscar. Densities of *L*. *catta* in Berenty surpass those observed in the Beza Mahafaly Special Reserve (216 individuals/km^2^) [70]. Likewise, densities of *P*. *verreauxi* were higher when compared with those in both the Vohidava-Betsimilaho Protected Area (96 individuals/km^2^) and the Beza Mahafaly Special Reserve (357individuals/km^2^) [70, 71]. Such numbers highlight the importance of Berenty as significant lemur conservation area in southern Madagascar.

Berenty Reserve is only one of the few remaining places in Madagascar that protect gallery and spiny forest sites. While it is also one of the relatively more studied ones in terms of plant composition and forest structure, many aspects of its plant diversity, function, and long-term forest dynamics remain unexplored. This gap in our knowledge is particularly marked in the spiny forest. Unfortunately, this lack of information extends to most Madagascar, with spiny and gallery forests being the least studied and protected ones [5, 9]. There is a need to expand our understanding of gallery and dry forests in Madagascar, and our study serves as a reference to compare to and replicate in other areas in the island.

### 4.6. Caveats and limitations

Despite the comprehensive analysis of Berenty Reserve’s unique ecosystems, this study’s scope and methodologies have inherent limitations. The differential sampling effort reflected in the number of plots examined in the spiny (6 plots) versus gallery forests (34 plots) may have biased our biodiversity comparisons. Similarly, our DBH threshold for tree inclusion may have excluded important tree species. Additionally, our study explored habitat quality for lemurs primarily through the lens of tree basal area and dietary preferences. While this approach offers important insights, a more comprehensive examination of lemur habitat requirements, including forest structure, annual variations in food availability and the impact of human disturbances, would provide a comprehensive understanding of habitat suitability and conservation needs [72]. Despite these caveats, our data provide strong support for the differentiated conservation strategies required for gallery and spiny forests in Madagascar and represents a valuable contribution to the expanding body of knowledge on the dry forest ecosystems of the island.

## Supporting information

S1 Fig Number of studies per research topic done in seven dry forest sites in Madagascar. Research papers about lemurs were overall the most numerous. The bars represent 518 journal articles found through the Web of Science using the site name + Madagascar as search words. Studies shown were conducted in the seven largest and most representative protected areas located in dry forests in Madagascar. Each article was assigned to one of the following categories, depending on the main area of research of the paper: Lemurs, lemurs’ interaction with plants (Lemurs/plants), forest (botany, ecology), mammals, insects, amphibians, birds, fossils, fungi, water quality, anthropology, sociology and socioecology.
(TIF)

S2 Fig Rarefaction and extrapolation curves for the spiny and gallery forests. Solid lines represent rarefaction curves, indicating observed species diversity, while dashed lines represent extrapolation beyond the sampled data, with the shaded areas showing confidence intervals.
(TIF)

S3 Fig Differences in species composition between forest types and forest sites. The differences were assessed using a non-metric multidimensional scaling ordination; a) gallery and spiny forest plots and b) three gallery forest sites. Distances between plots (circles, squares, or triangles) are proportional to the differences in species composition. The plot only shows species with a p-value ≤ 0.001 (Celgom: *Celtis gomphophylla*; Comlam: *Commiphora lamii*; Comhum: *Commiphora humbertii*; Corsin: *Cordia sinensis*; Ecfmai: *Euphorbia cf*. *mainty*; Gyrame: *Gyrocarpus americanus*; Pitdul: *Pithecellobium dulce*; Salang: *Salvadora angustifolia*; Grecal: *Grewia calvata*).
(TIF)

S4 Fig Importance Value Index (IVI_f_) of the plant families in Berenty Reserve, Madagascar. The IVI_f_ pertains to **(a)** the gallery and **(b)** spiny forests. Additionally, the three forest sites within the gallery forests are shown: **(c)** Anaramalangy, **(d)** Ankoba, and **(e)** Malaza. According to the IVI_f_, the five most important families were Fabaceae (73.8), Violaceae (61.5), Salvadoraceae (24.8), Capparaceae (23.1), and Cannabaceae (20.5). The top five families accounted for 67% of the overall IVI_f_. However, the five most important families differed between the gallery and the spiny forests. In the gallery forest, the most important families were Fabaceae (68.6), Violaceae (61.9) Cannabaceae (33.3), Salvadoraceae (30.5), and Capparaceae (25.7). In the spiny forest the top 5 were Didieraceae (129.4), Burseraceae (51.9), Malvaceae (28.0), Hernandiaceae (15.5), and Bignoniaceae (14.6) (Fig 6). Likewise, the five most important families differed among the three gallery forest sites. For Ankoba these were Fabaceae (111.50), Cannabaceae (52.80), Violaceae (43.68), Boraginaceae (19.88) and Capparaceae (13.72). In Malaza, they were Violaceae (44.6), Fabaceae (7.32), Salvadoraceae (8.32), and Capparaceae (8.17). In Anaramalangy, these were Fabaceae (10.38), Salvadoraceae (4.18), Violaceae (2.54), Capparaceae (2.54) and Malvaceae (2.24).
(TIF)

S5 Fig Frequency distribution of tree diameter size classes in the gallery and spiny forests in Berenty Reserve. To enhance visibility of smaller values the total number of individuals was log transformed.
(TIF)

S6 Fig Frequency distribution of tree diameter size classes in the three gallery forest sites in Berenty Reserve. To enhance visibility of smaller values the total number of individuals was log transformed.
(TIF)

S7 Fig db-RDA ordination of the plots constrained by environmental variables. DistanceBased Redundancy Analysis (db-RDA) was conducted to explore the relationship between soil microbial communities and environmental variables in **(a)** the spiny and gallery forest plots, and **(b)** Anaramalangy, Ankoba, and Malaza forest plots in the gallery forest. Each dot represents a plot that has been color-coded by forest type or forest site. The proximity of points indicates similarity in plant community composition. Arrows indicate the significant explanatory environmental variables.
(TIF)

S8 Fig Tamarind density in the different gallery forest plots sampled in Berenty Reserve. **(a)** Density of tamarind seedlings found in the subplots. All the tamarind (%) individuals in the subplots had a diameter at root collar ≤ 1 cm, so were therefore classified as seedlings **(b)** Density of adult individuals found in the 0.1 ha plots, with a DBH ≥ 10 cm.
(TIF)

S9 Fig Relationship between tamarind seedling density and adult seedling density in each of the gallery forest plots in Berenty Reserve. Each dot represents one of the plots in the gallery forest (n = 34).
(TIF)

S10 Fig Lemur habitat quality of the gallery and spiny forests in Berenty Reserve. The habitat quality is shown as **(a)** the total basal area of the primary tree species constituting >75% of the lemur diet, **(b)** the relative basal area of all food tree species consumed by the three species of diurnal lemurs, and **(c)** the relative basal area of the primary tree species constituting >75% of the lemur diet.
(TIF)

S11 Fig Lemur habitat quality of the gallery forest sites in Berenty Reserve. Habitat quality is represented as (a) the total basal area of the primary tree species constituting >75% of the lemur diet, (b) the relative basal area of all food tree species consumed by the three species of diurnal lemurs, and (c) the relative basal area of the primary tree species constituting >75% of the lemur diet.
(TIF)

S1 Table Number of surveyed plots in Berenty Reserve. Plots are classified by forest type and forest site.
(XLSX)

S2 Table List of species present in the spiny and gallery forest at Berenty Reserve. Species are classified as endemic and/or native, and their threat category from the IUCN is also indicated.
(XLSX)

S3 Table Abundance and density of the ring-tailed (*L*. *catta*) and the sifaka (*P*. *verreauxi*) lemurs in different spiny and gallery forest sites in Berenty Reserve. Lemur abundances and densities were recorded in the year 2020 as part of an ongoing and long-term lemur census in Berenty Reserve [57].
(XLSX)
