# Supplementary figures and images for: Structural and compositional differences in gallery and spiny forests of Southern Madagascar: Implications for conservation of lemur and tree species
Source: PLoS One. 2024 Aug 29;19(8):e0307907. doi: 10.1371/journal.pone.0307907 (PMC11361438; doi:10.1371/journal.pone.0307907)

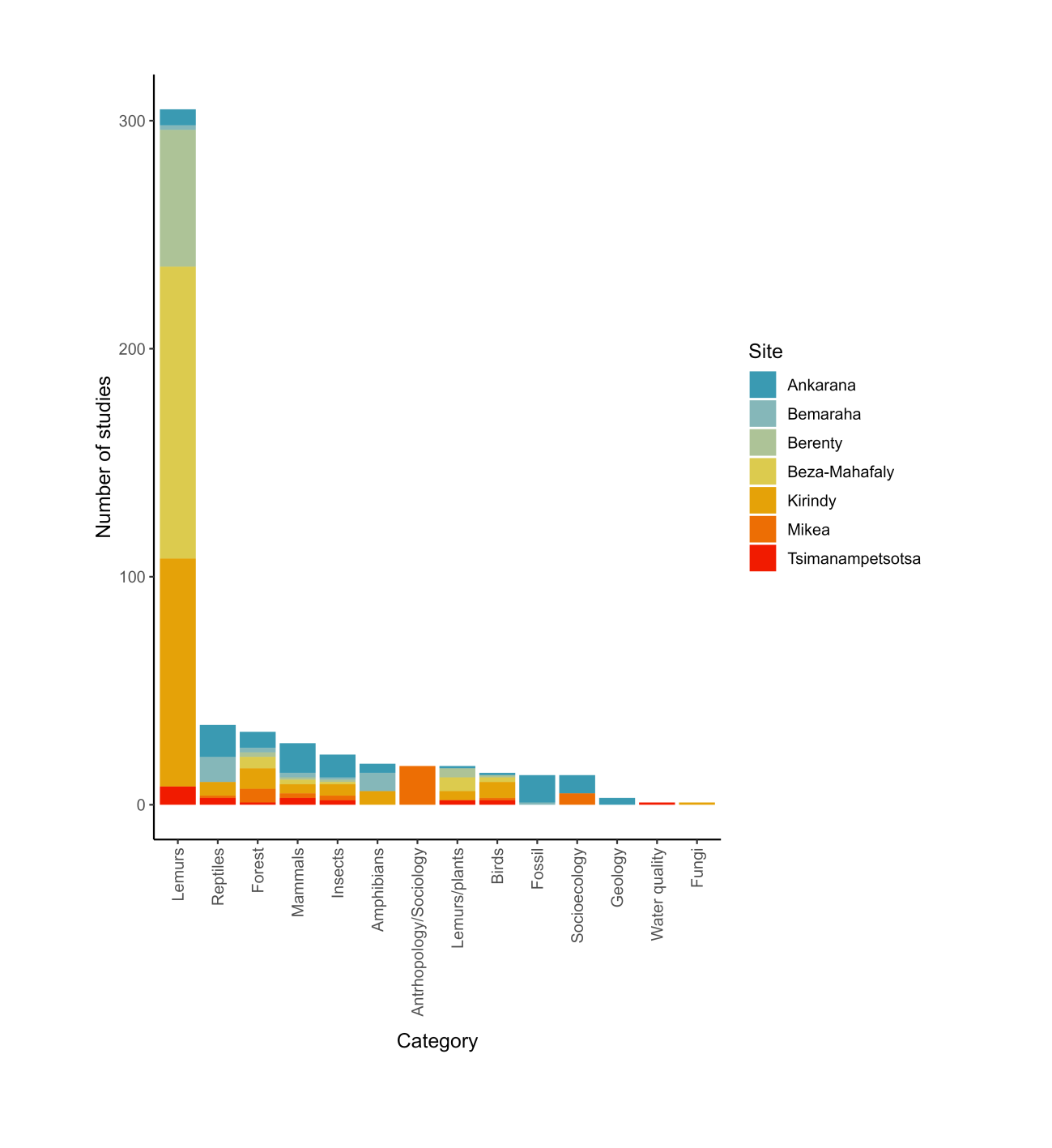

Supplement: S1 Fig — Research papers about lemurs were overall the most numerous. The bars represent 518 journal articles found through the Web of Science using the site name + Madagascar as search words. Studies shown were conducted in the seven largest and most representative protected areas located in dry forests in Madagascar. Each article was assigned to one of the following categories, depending on the main area of research of the paper: Lemurs, lemurs’ interaction with plants (Lemurs/plants), forest (botany, ecology), mammals, insects, amphibians, birds, fossils, fungi, water quality, anthropology, sociology and socioecology. (TIF) [file pone.0307907.s001.tif]

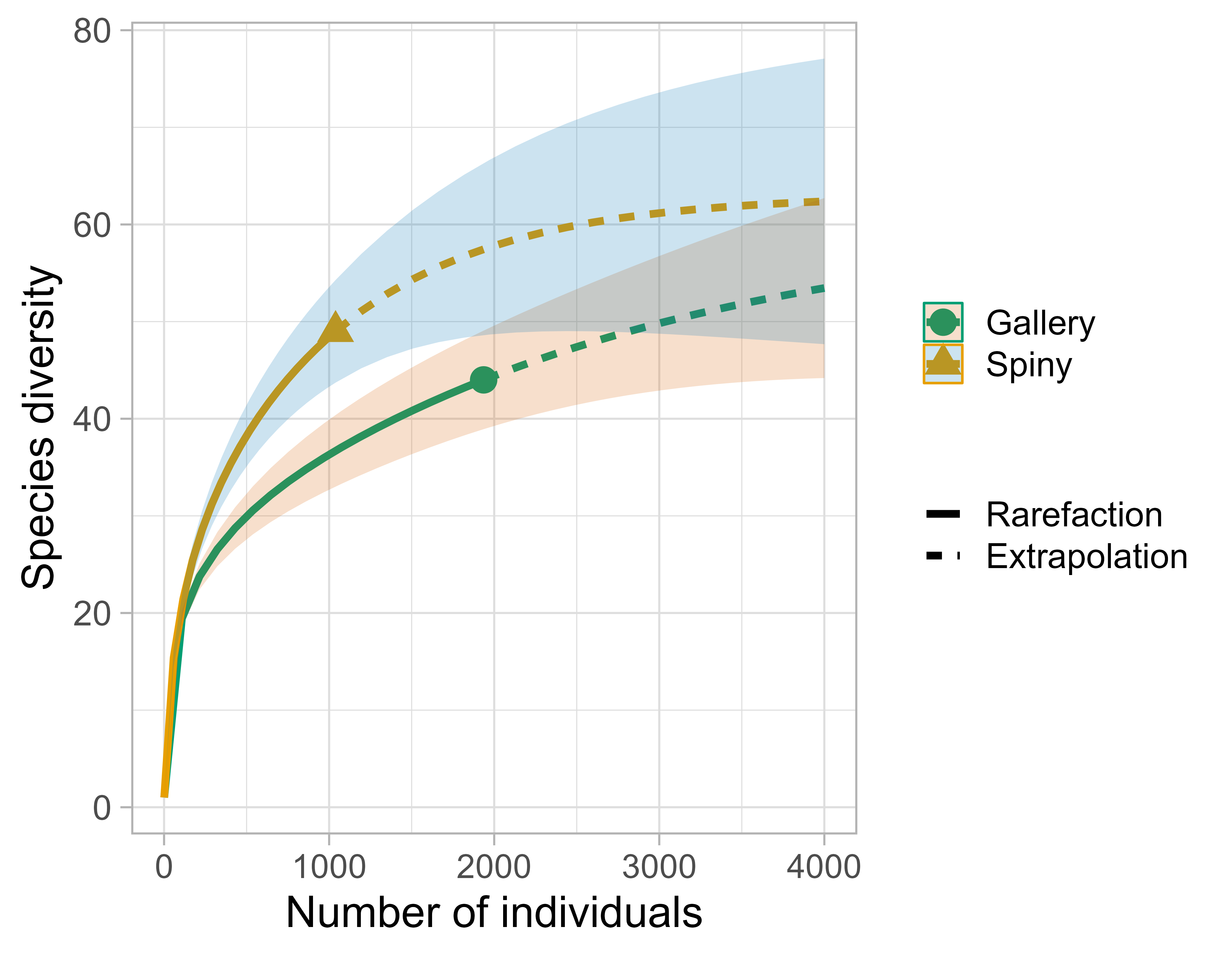

Supplement: S2 Fig — Solid lines represent rarefaction curves, indicating observed species diversity, while dashed lines represent extrapolation beyond the sampled data, with the shaded areas showing confidence intervals. (TIF) [file pone.0307907.s002.tif]

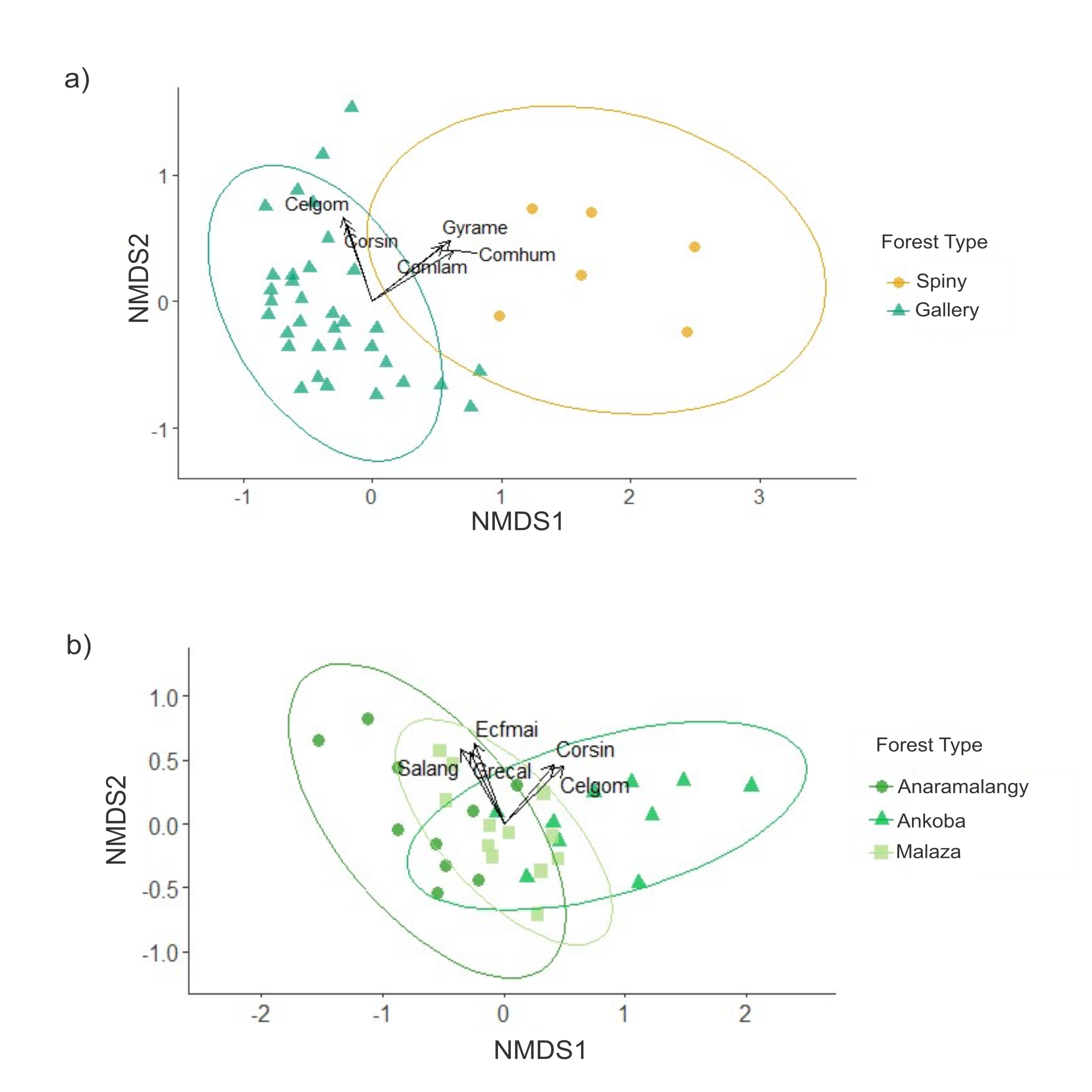

Supplement: S3 Fig — The differences were assessed using a non-metric multidimensional scaling ordination; a) gallery and spiny forest plots and b) three gallery forest sites. Distances between plots (circles, squares, or triangles) are proportional to the differences in species composition. The plot only shows species with a p-value ≤ 0.001 (Celgom: Celtis gomphophylla; Comlam: Commiphora lamii; Comhum: Commiphora humbertii; Corsin: Cordia sinensis; Ecfmai: Euphorbia cf. mainty; Gyrame: Gyrocarpus americanus; Pitdul: Pithecellobium dulce; Salang: Salvadora angustifolia; Grecal: Grewia calvata). (TIF) [file pone.0307907.s003.tif]

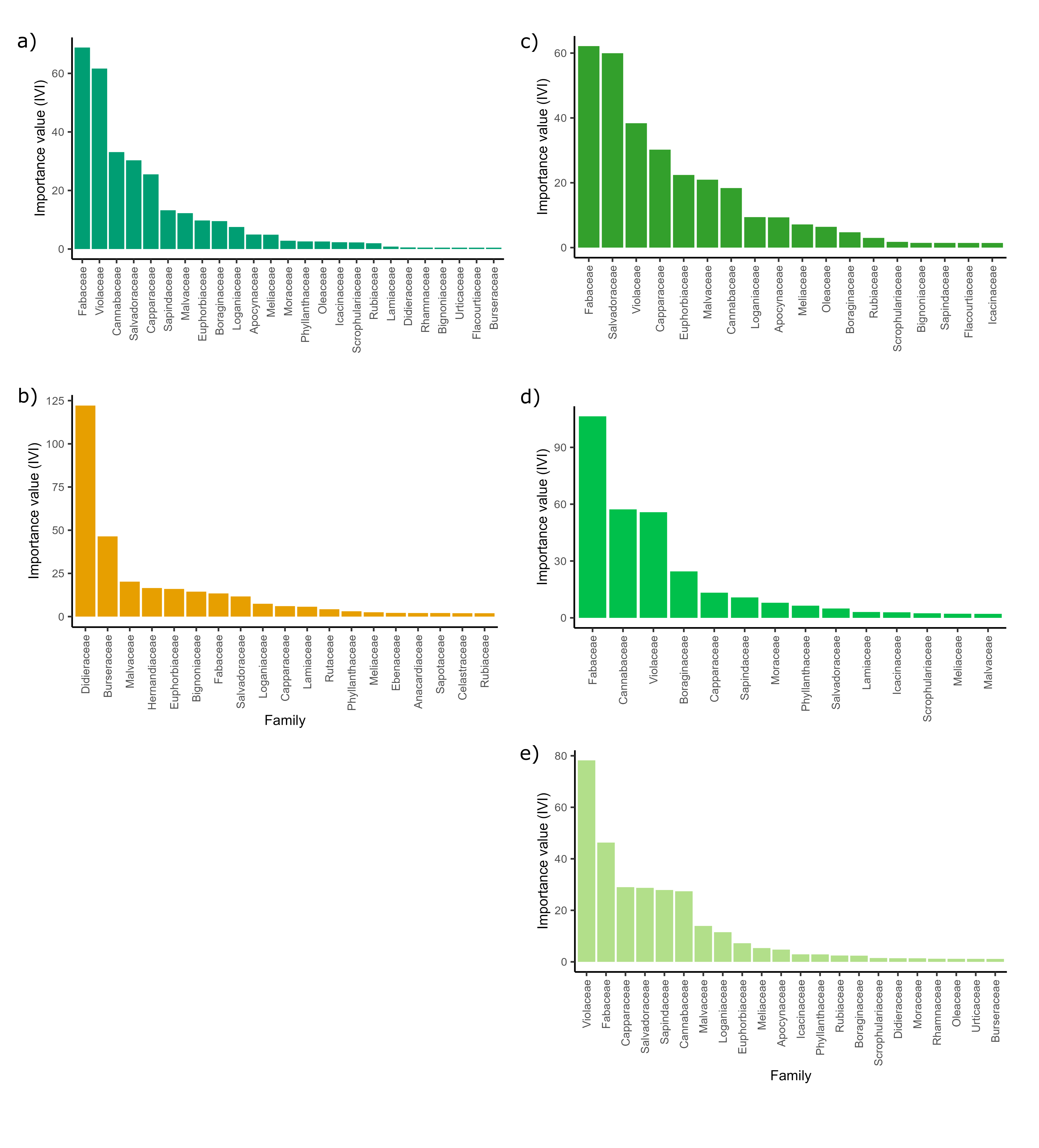

Supplement: S4 Fig — The IVIf pertains to (a) the gallery and (b) spiny forests. Additionally, the three forest sites within the gallery forests are shown: (c) Anaramalangy, (d) Ankoba, and (e) Malaza. According to the IVIf, the five most important families were Fabaceae (73.8), Violaceae (61.5), Salvadoraceae (24.8), Capparaceae (23.1), and Cannabaceae (20.5). The top five families accounted for 67% of the overall IVIf. However, the five most important families differed between the gallery and the spiny forests. In the gallery forest, the most important families were Fabaceae (68.6), Violaceae (61.9) Cannabaceae (33.3), Salvadoraceae (30.5), and Capparaceae (25.7). In the spiny forest the top 5 were Didieraceae (129.4), Burseraceae (51.9), Malvaceae (28.0), Hernandiaceae (15.5), and Bignoniaceae (14.6) (Fig 6). Likewise, the five most important families differed among the three gallery forest sites. For Ankoba these were Fabaceae (111.50), Cannabaceae (52.80), Violaceae (43.68), Boraginaceae (19.88) and Capparaceae (13.72). In Malaza, they were Violaceae (44.6), Fabaceae (7.32), Salvadoraceae (8.32), and Capparaceae (8.17). In Anaramalangy, these were Fabaceae (10.38), Salvadoraceae (4.18), Violaceae (2.54), Capparaceae (2.54) and Malvaceae (2.24). (TIF) [file pone.0307907.s004.tif]

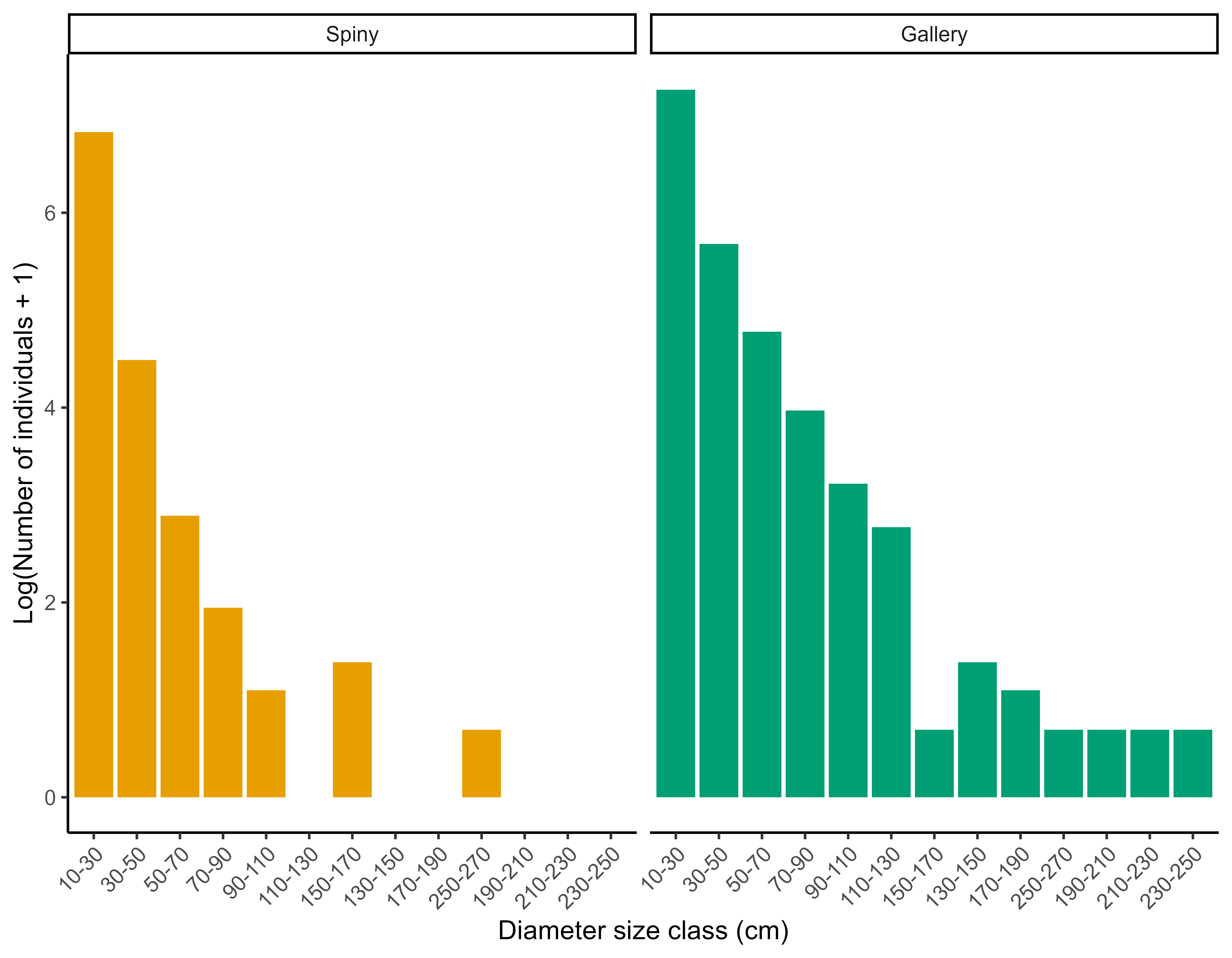

Supplement: S5 Fig — To enhance visibility of smaller values the total number of individuals was log transformed. (TIF) [file pone.0307907.s005.tif]

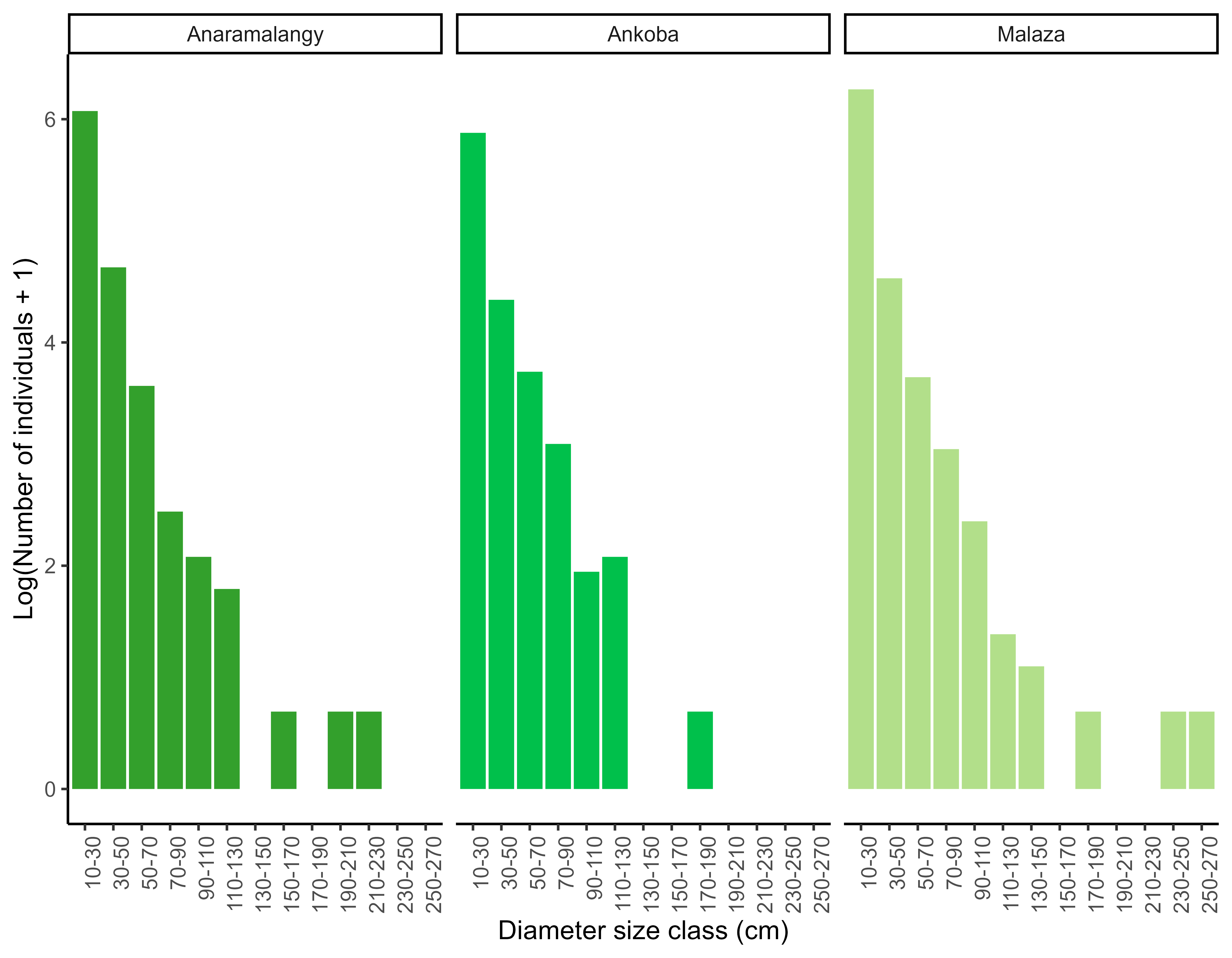

Supplement: S6 Fig — To enhance visibility of smaller values the total number of individuals was log transformed. (TIF) [file pone.0307907.s006.tif]

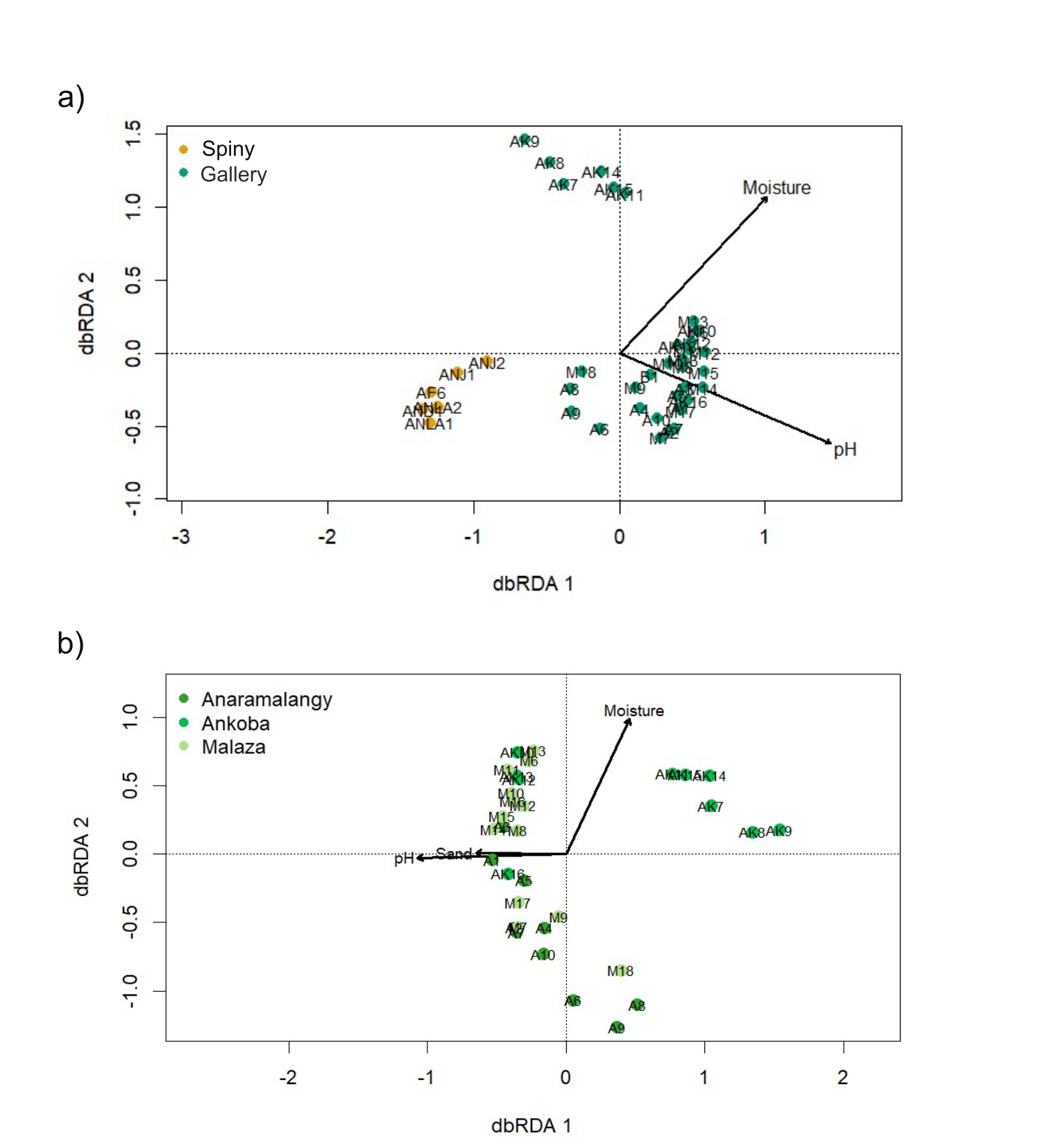

Supplement: S7 Fig — DistanceBased Redundancy Analysis (db-RDA) was conducted to explore the relationship between soil microbial communities and environmental variables in (a) the spiny and gallery forest plots, and (b) Anaramalangy, Ankoba, and Malaza forest plots in the gallery forest. Each dot represents a plot that has been color-coded by forest type or forest site. The proximity of points indicates similarity in plant community composition. Arrows indicate the significant explanatory environmental variables. (TIF) [file pone.0307907.s007.tif]

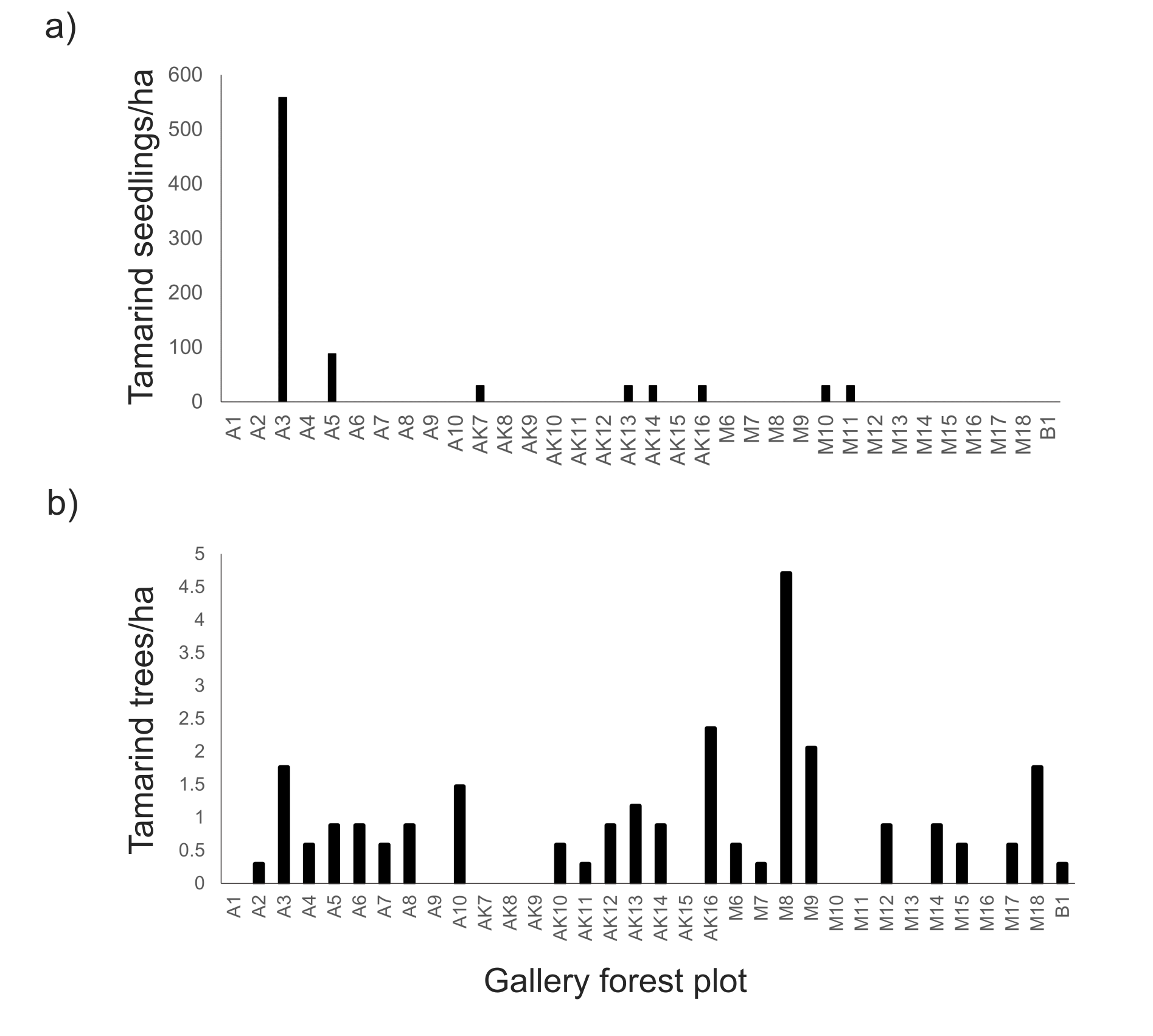

Supplement: S8 Fig — (a) Density of tamarind seedlings found in the subplots. All the tamarind (%) individuals in the subplots had a diameter at root collar ≤ 1 cm, so were therefore classified as seedlings (b) Density of adult individuals found in the 0.1 ha plots, with a DBH ≥ 10 cm. (TIF) [file pone.0307907.s008.tif]

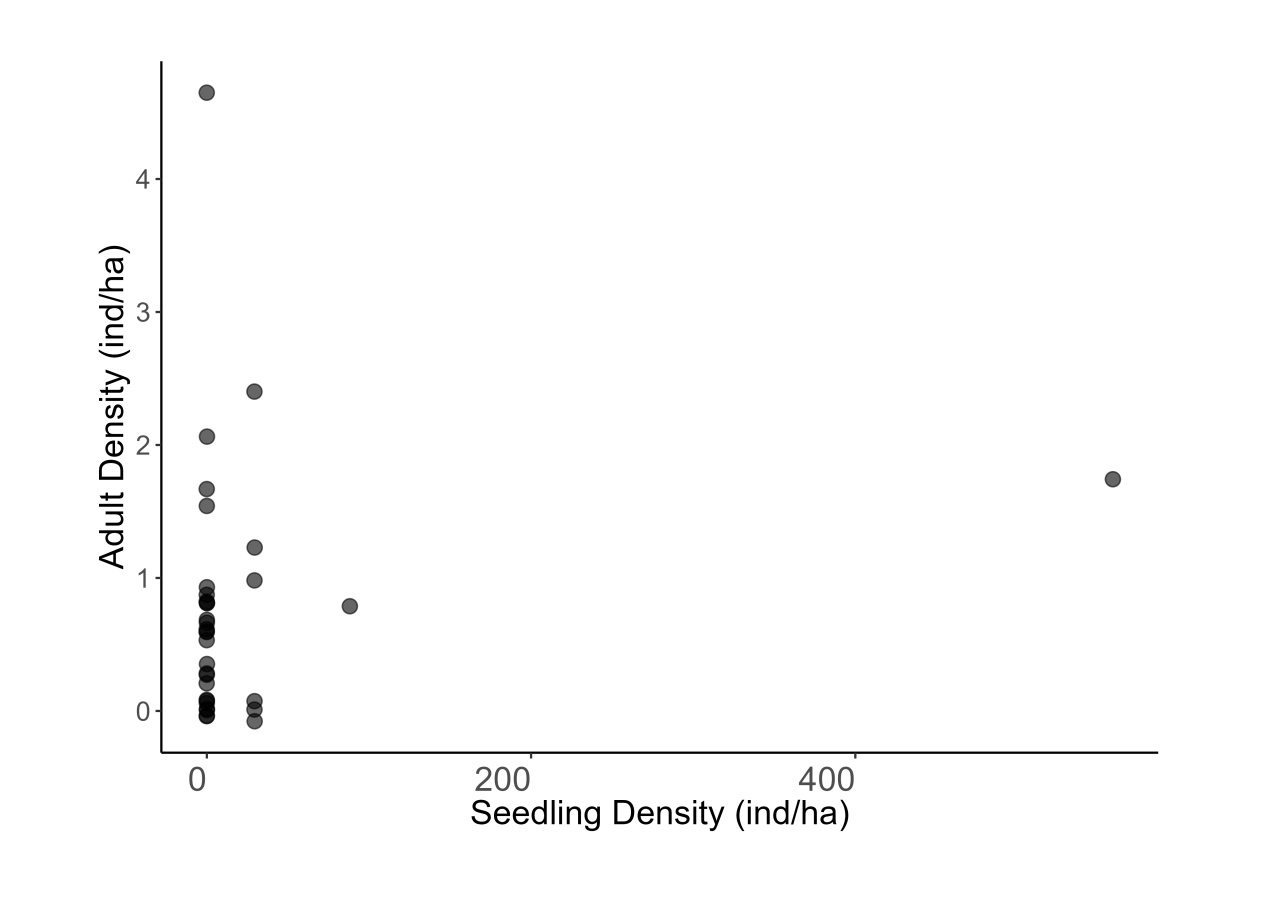

Supplement: S9 Fig — Each dot represents one of the plots in the gallery forest (n = 34). (TIF) [file pone.0307907.s009.tif]

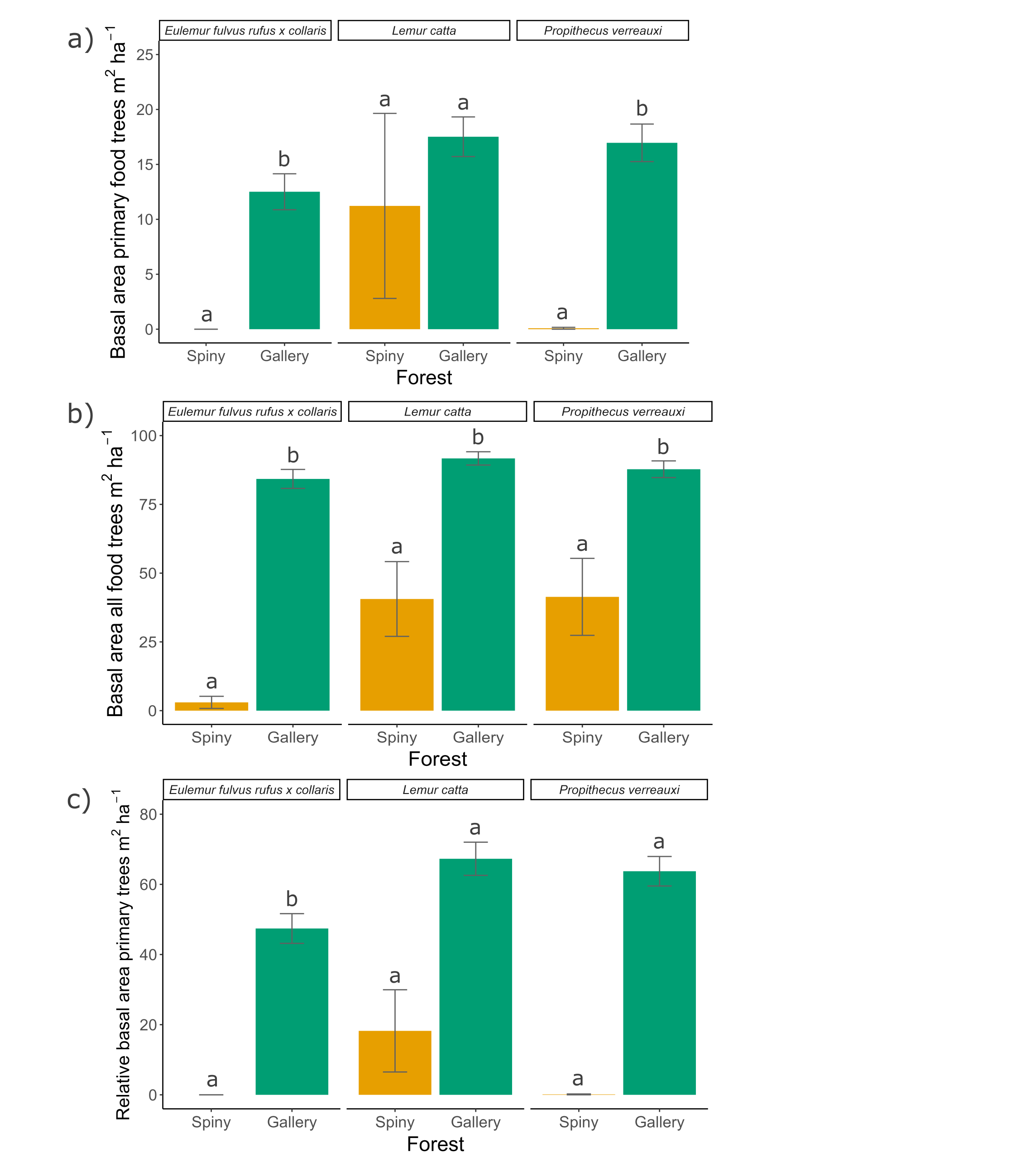

Supplement: S10 Fig — The habitat quality is shown as (a) the total basal area of the primary tree species constituting >75% of the lemur diet, (b) the relative basal area of all food tree species consumed by the three species of diurnal lemurs, and (c) the relative basal area of the primary tree species constituting >75% of the lemur diet. (TIF) [file pone.0307907.s010.tif]

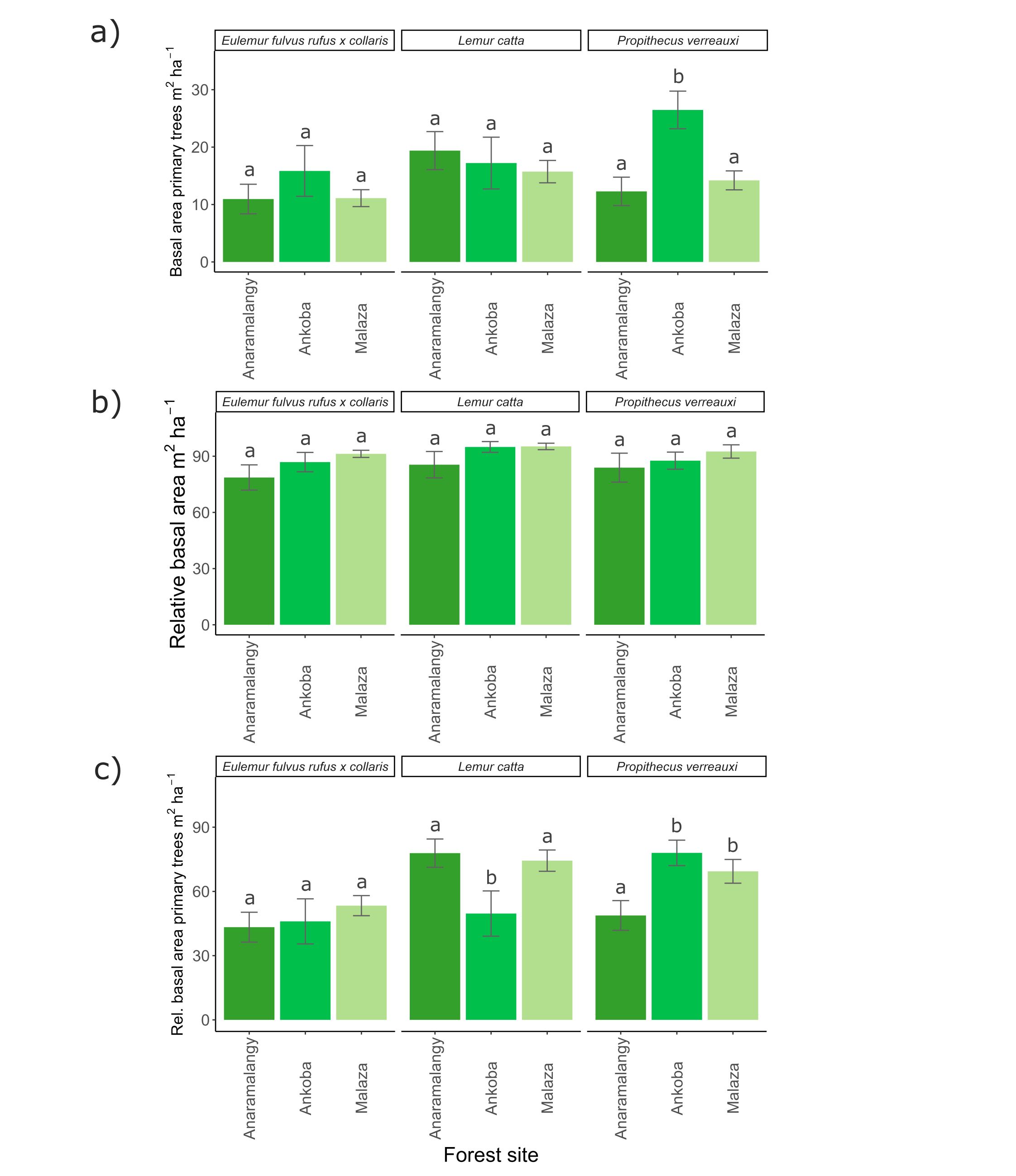

Supplement: S11 Fig — Habitat quality is represented as (a) the total basal area of the primary tree species constituting >75% of the lemur diet, (b) the relative basal area of all food tree species consumed by the three species of diurnal lemurs, and (c) the relative basal area of the primary tree species constituting >75% of the lemur diet. (TIF) [file pone.0307907.s011.tif]
